# Patients’ perspectives on medication adherence feedback interventions for managing long-term medications: a systematic review of qualitative evidence

**DOI:** 10.1007/s11096-025-01958-4

**Published:** 2025-07-16

**Authors:** Barbara Kobson, Janet Hanley, Alpana Mair, Alexandra L Dima, Nicola Rea, Ruth E Paterson

**Affiliations:** 1 Edinburgh Napier University, Edinburgh, United Kingdom of Great Britain and Northern Ireland; 2 Avedis Donabedian Research Institute (FAD) - Universitat Autònoma de Barcelona (UAB), Barcelona, Spain; 3 Avaluació de tecnologies sanitàries en atenció primària i salut mental (PRISMA), Institut de Recerca Sant Joan de Déu (IRSJD) - Consortium “Centro de Investigación Biomédica en Red” Epidemiology and Public Health (CIBERESP), Esplugues de Llobregat, Spain

**Keywords:** Feedback interventions, Long-term medication, Medication adherence, Patient perspectives

## Abstract

**Introduction:**

Optimising medication usage is a worldwide challenge. While numerous feedback interventions have been developed to address this issue, understanding patients' perspectives on the use of such interventions to optimise adherence provides opportunities for successful development and implementation.

**Aim:**

To synthesise qualitative evidence on patients' views on medication adherence feedback interventions to support adherence behaviour.

**Method:**

CINAHL, EMBASE, MEDLINE, PsycINFO, and PubMed were systematically searched from database inception to February 2023 with searches updated to February 2025. Additionally, Google Scholar was used to identify any potentially relevant grey literature or supplementary sources. Eligible studies included qualitative or mixed-methods research that explored adult patients’ perspectives on medication adherence feedback interventions for long-term conditions, specifically those aimed at self-management within community settings. The review was conducted according to ENTREQ and reported following PRISMA guidelines. Study quality was appraised using the Mixed Methods Appraisal Tool (MMAT). Data were extracted and analysed using thematic synthesis, with findings presented narratively.

**Results:**

Of the 1270 studies screened, 11 met the inclusion criteria and evaluated participants’ views on therapeutic drug monitoring and digital adherence interventions across conditions including asthma, human immunodeficiency virus (HIV), coronary heart disease, hypertension, type 2 diabetes, and opioid use disorder. Three themes were identified; balancing support and autonomy in feedback interventions, maintaining patient-provider relationship and enhancing engagement through tailored design. Interventions were considered acceptable when they were easy to use, offered users control over personal data, incorporated audio-visual cues, and provided emotional or motivational support. Trust and shared decision-making between patients and providers facilitated uptake, while tailored interventions were considered essential for supporting engagement.

**Conclusion:**

Medication adherence feedback interventions are acceptable, however further improvements will enhance user engagement and optimise adherence. Future research should prioritise co-designed interventions that address user needs, improve patient-provider communication, deliver accurate adherence feedback, and support cost-effective scalability.

**Supplementary Information:**

The online version contains supplementary material available at 10.1007/s11096-025-01958-4.

## Impact statements

- Involving patients in medication adherence intervention development would promote engagement with the intervention and optimise adherence.
- Establishing good relationships between patients and healthcare providers would enhance shared decision-making and promote effective self-management of prescribed medications.
- Providing accurate, real-time feedback on patients’ medication adherence through visually presented objective data can enhance understanding of adherence behaviours and support problem-solving efforts.
- Assessing medication adherence levels based on prescribing and dispensing data from electronic health records could be more accessible, cost effective and enhance scalability across wider patient populations.

## Introduction

Medication adherence is an important behaviour for optimising health [1]. It is defined as ‘the process by which patients take their medication as prescribed, divided into three phases; initiation, implementation and discontinuation’ [2]. Although benefits for people living with chronic conditions are well established, medication adherence remains a global problem with approximately 50% of patients not adhering to medications [3–5], leading to poor outcomes and increased healthcare cost [6].

Low adherence to medication increases adverse health outcomes including mortality and hospitalisation [7, 8]. A meta-analysis evaluating the impact of adherence in coronary artery disease patients associated poor adherence with a higher risk of all-cause mortality and cardiovascular hospitalisation [9]. Similarly, a systematic review of twenty-three studies assessing correlations between non-adherence to diabetes medications and clinical outcomes reported suboptimal control of blood glucose levels increased the risk of diabetic complications [10]. This evidence suggests a need for adequate adherence support to improve clinical outcomes.

Both digital and non-digital solutions have been developed over the years to address medication non-adherence [11]. A systematic review of systematic reviews by Anderson et al. [12] found that dose simplification, education, electronic reminders, and financial incentives are effective for improving adherence. Additionally, the rapid evolution of digital health information technology has led to electronic health interventions being more widely accessible to support medication adherence [11, 13] albeit complex and with modest effectiveness [14, 15]. Among these, adherence monitoring and feedback strategies including pill counts, pharmacy refills, electronic and therapeutic drug monitoring systems have shown promise in improving adherence [16–18]. This outcome could be explained by behavioural theories including Bandura’s social cognitive theory of self-regulation which posits that providing feedback on adherence behaviour increases awareness and facilitates goal setting and planning to achieve adherence [19]. Hence, underpinning interventions with self-regulation components could improve adherence and should be considered in design and developmental phases. eHealth feedback interventions, however, have their limitations. Concerns with data accuracy, cost and user burden influence acceptability and scalability to the wider population [20, 21]. However, improvement efforts have leveraged prescribing and dispensing data from electronic health records (EHR) to assess and provide feedback on adherence [22]. This approach addresses certain limitations and promotes a person-centred strategy to optimising medication use [23].

While the effectiveness of feedback interventions has been extensively studied [24–27], limited research has explored patients' perspectives on these approaches. As policymakers increasingly recognise the value of person-centred care in empowering patients [28], future studies should prioritise understanding user-experience. Such insights would inform the development of interventions that are both effective and aligned with user needs.

### Aim

The aim of our review was to synthesise qualitative evidence on patients' views of medication adherence feedback interventions to support adherence behaviour.

## Method

### Synthesis methodology

Our review was underpinned by Thomas and Harden’s [29] methodology which recommends a structured approach to synthesising qualitative evidence. The Preferred Reporting Items for Systematic Reviews and Meta-Analyses (PRISMA) Checklist [30] and ‘enhancing transparency in reporting the synthesis of qualitative research’ (ENTREQ) [31] guidelines were used to direct the reporting and conducting of this systematic review, respectively. The review was registered prospectively in PROSPERO (CRD42023434755).

### Search strategy

Five bibliographic database sources including Cumulative Index of Nursing and Allied Health Literature (CINAHL), MEDLINE, EMBASE, PsycINFO, and PubMED were searched for relevant studies. Google Scholar was searched to identify relevant citations and EThOS (Electronic Theses Online Service) [32] was also searched for access to published theses that would be relevant for this review. Reference lists from the identified studies were also searched to facilitate a comprehensive process for obtaining the relevant papers. Key search terms including feedback interventions, patient perspectives, and medication adherence were applied as medical subject heading (MeSH) terminology or used as key words (Supplementary file 1). Eligible studies were included from database inception to February 2023. The search was updated to identify new studies between February 2023 and February 2025.

### Screening and study selection methods

Records were initially screened by their titles and abstracts then by full texts, using pre-determined inclusion and exclusion criteria (Table 1). Two reviewers (BK and NR) completed the screening and selection process, and a third reviewer (RP) resolved any difference.

**Table 1:** Inclusion and exclusion criteria

*Inclusion criteria*
Participants on long-term medication for chronic conditions, aged 18 and above Interventions providing adherence feedback for medication self-management Qualitative studies/ mixed methods studies/ quantitative surveys with qualitative components Studies reporting on participants’ perspectives of medication adherence feedback interventions Primary research Published and grey literature
*Exclusion criteria*
Studies involving participants with mental health conditions Protocols Conference papers Articles without full texts

### Quality assessment

Quality appraisal was undertaken using the Mixed Methods Appraisal Tool (MMAT) [33], which was selected due to its suitability for assessing the methodological quality of studies with diverse designs, including qualitative, quantitative, and mixed-methods approaches. This allowed a consistent framework for evaluating all included studies. One reviewer (BK) conducted the primary appraisal of each study, while a second reviewer (RP) independently checked all appraisals to ensure accuracy and reduce bias. Any discrepancies were resolved through discussion. Colour codes were employed to visually represent the response to each MMAT criterion: green indicated "yes", yellow signified "can't tell", and red denoted "no".

### Data extraction

A data extraction tool was developed using a bespoke table to extract essential information from the included studies. Study characteristics including author, year of publication, study setting, location and findings were extracted from each study and presented in the table.

### Data synthesis

Each study was imported as a PDF and stored within the NVivo 20 software. A thematic synthesis approach as described by Thomas and Harden [29] was applied after relevant codes from the results and discussion sections of the included studies had been extracted. This approach involved free line-by-line coding, developing descriptive themes and generating analytical themes. Initial sets of codes were identified from one paper and used as a coding framework to which other codes from subsequent papers were added, generating 125 codes. Coding was done inductively and completed by one reviewer (BK). Four other reviewers (RP, JH, AM, and AD) assessed the coding process and consistency between the developed codes and the data obtained from included studies. Texts from the original studies were revisited, to ensure coherence and confirm the codes reflected the views and experiences expressed by study participants. Descriptive themes were developed and analysed to generate analytical themes.

## Results

### Study selection

**Fig. 1:**
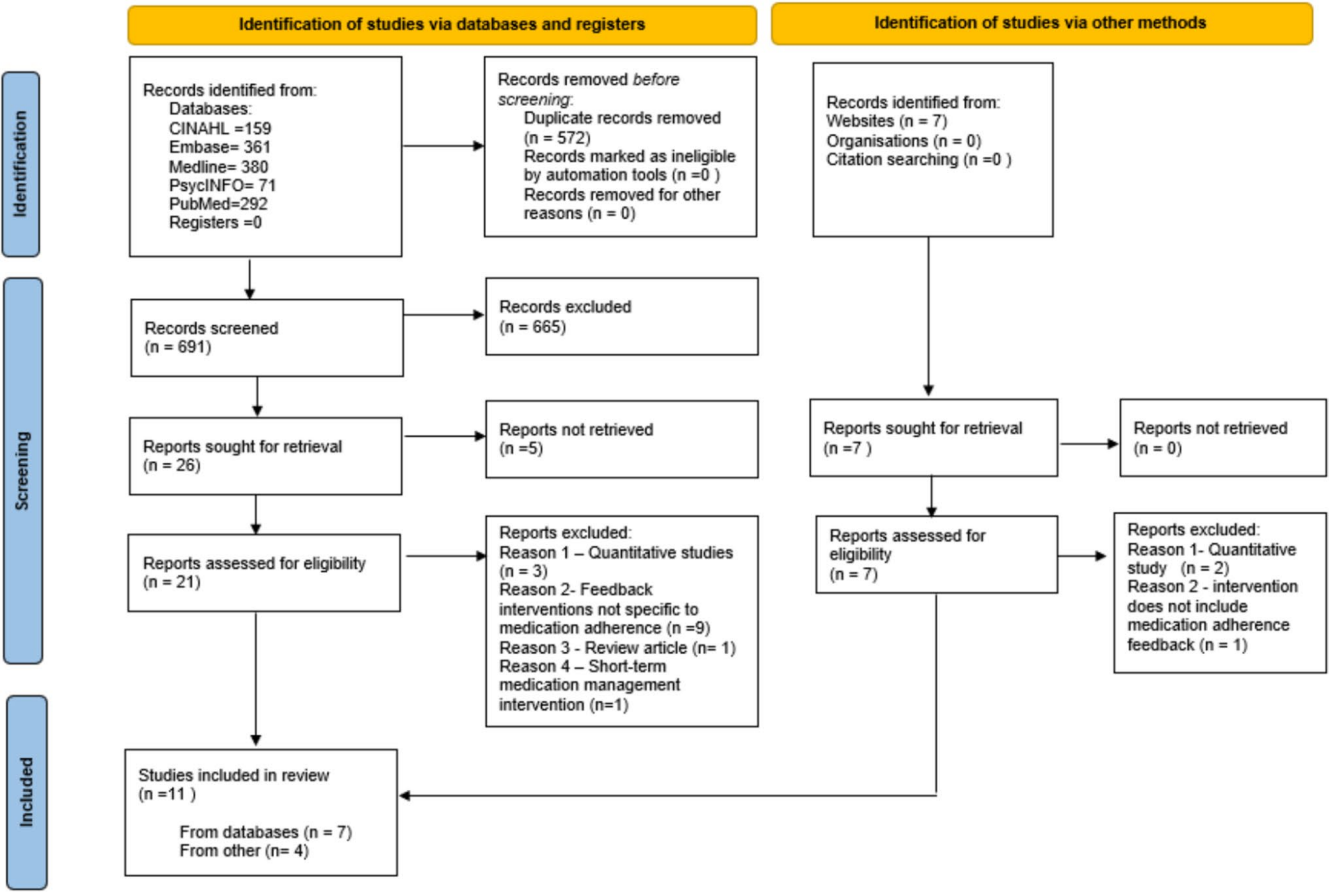
PRISMA flow diagram depicting included and excluded articles (Updated search: February 2023–February 2025)

A total of 1270 records were retrieved from electronic databases and Google Scholar following an initial screening of titles and abstracts. Twenty-eight studies were eligible for full-text screening of which 11 met the inclusion criteria and were selected for the review. Participants from the included studies were 330 in total with diagnoses including human-immunodeficiency virus (HIV), asthma, coronary artery disease, type 2 diabetes, hypertension and opioid use disorder. Figure 1 illustrates the flow diagram of the screening and selection process, while study characteristics are summarised in Table 2.

**Table 2:** Characteristics of included studies

References, Country	Study aim	Study design/data collection method	Participant characteristics	Data analysis	Intervention/component	Summary of findings
Nelson et al. [34] United States	To understand how people with Type 2 diabetes mellitus (T2DM) experience a text messaging and interactive voice response (IVR) delivered medication adherence intervention	Study design: mixed methods study Data collection method: Questionnaires	Sample size: n = 80 Patient group: Type 2 diabetes Age (mean): 50.0 ± 10.1 years Sex: male ( 35%) Setting: health centre	Content analysis	Intervention: Text messaging service with interactive voice response for T2DM patients Component: Daily text messages to address one barrier Daily text messages to assess adherence Weekly interactive voice response feature	Young and newly diagnosed patients benefited from the intervention Daily text messages were useful Daily texts preferred over weekly IVR
Kuipers et al. [35] Netherlands	To assess validity and patient-reported usability and acceptability of an electronic adherence monitor and reminder device for patient care and research	Study design: Mixed methods study Data collection method: Questionnaires; open ended and closed questions	Sample size: n = 32 Patient group: asthma patients Age (mean): 48.1 years Sex: male (47%) Setting: community pharmacies	Grounded theory and descriptive statistics	Intervention: Inhalation monitoring using an electronic inhalation medication device (EIMD) Component: Electronic inhaler device Mobile phone app Online portal	App was easy to use App was useful but participants were not willing to continue use after study EMID should be tailored to participants’ needs and preferences
Pack et al. [36] United States	To develop three graphical display prototypes of hypothetical daily concentrations of ART measured in hair and assess their acceptability among participants	Study Design: Qualitative study Data collection method: Interviews	Sample size: n = 30 Patient group: HIV positive patients Age:18 years or older Sex: male (70%) Setting: university based infectious disease clinic	Framework analysis	Intervention: Therapeutic drug monitoring in hair Component: Graphical display prototypes of hair MSI results (pictographs, bar, and line graphs)	Visual data improves cognition of health data Pictographs preferred over bar graphs in people with lower health literacy Graphical displays to have both patient and clinician versions to enhance understanding
Schesing et al. [37] United States	To assess attitudes of patients and physicians towards therapeutic drug monitoring and the potential impact on physician and patient relationship	Study Design: Qualitative study Data collection method: semi-structure interviews	Sample size: n = 11 Patient group: hypertensive patients on low income Age (mean): 62 ± 11 years Sex: male (27%) Setting: general medicine and cardiology clinics	Thematic content analysis	Intervention: Therapeutic drug monitoring (TDM) Component: TDM guided feedback (blood test results)	Feedback interventions found to be helpful Interventions could negatively impact patients and providers relationships Non-judgemental feedback approach preferred by patients
Faisal et al. [38] Canada	To assess stakeholders’ feedback and experience on using electronic medication adherence products	Study design: qualitative study Data collection method: interviews	Sample size: n = 21 Patient group: older adults 65y or older taking one or more medications Age (mean): 75 years Sex: male (48%) Setting: research laboratory	Thematic analysis	Intervention: Electronic medication adherence products Component: Audio and visual reminders Medication tracking features and multi-compartmental products	Product and user factors affect user preference Continued engagement with products enhances familiarity Preference for products which allow complex medication regimens, are affordable, has reminder alarms, are portable and easy to use User characteristics should be considered when selecting products
Hill et al. [39] United States	To understand patients’ and Clinicians’ perspectives on objective feedback to enhance conversations about adherence	Study design: qualitative study Data collection method: Individual interviews (semi-structured)	Sample size: n = 30 Patient group: HIV positive patients Age: 18 years or older Sex: male (70%) Setting: infectious disease clinic	Thematic analysis	Intervention: Therapeutic drug monitoring in hair Component: Hair MSI results	Provides accurate real-time feedback on adherence Enhances problem solving Useful for both adherent and non-adherent patients Facilitates teachable moments on adherence Could lead to mistrust in the patient-clinician relationship Non-judgemental feedback approach preferred
Park et al. [40] United states	To explore coronary heart disease (CHD) patients’ perceptions, ideas, attitudes, and beliefs on the use of text messaging and mobile apps for medication adherence	Study design: Qualitative study Data collection method: Semi structured and open interview questions	Sample size: n = 28 Patient group: history of ACS/ PCI Age (median): 69.5 ± 10.8 years Sex: male (75%) Setting: cardiac conditioning centres and veteran affairs medical centres	Grounded theory analysis	Intervention: Medication reminder apps (Mango Health and Medisafe) to improve medication adherence Component: Text messaging features One or two-way reminders	Text messaging helpful as reminders for medication and clinic appointments and for establishing medication routines Frequent texts messages lead to message fatigue Preference for personalised text messages Users will be engaged if photos embedded within text messages Encouraging text messages can appear patronising Ability to interact with pill and appointment reminders was useful Having access to charts and graphs of medication adherence histories was helpful Preference for app data to be linked to healthcare systems
Ngowi et al. [41] Tanzania	To assess post intervention acceptability of digital adherence technology in participants who took part in the REMIND TRIAL	Study design: Qualitative study (nested within a 3-arm RCT) Data collection method: structured in-depth interviews	Sample size: n = 19 Patient group: HIV positive patients Age (median): 43 years Sex: male (37%) Setting: HIV care and recruitment centres	Thematic framework analysis	Intervention: Digital Adherence Tools (DATs) Component: Real-time medication monitoring (RTMM) and SMS text messaging service	SMS and real-time medication monitoring interventions were found acceptable Using graphs to describe adherence status was deemed acceptable DATs received a favourable opinion from users
Adejumo et al. [42] United Kingdom	To understand participants’ perceptions and experience of using electronic monitoring devices	Study design: Qualitative study Data collection method: Semi structured interviews	Sample size: n = 28 Patient group: asthma patients Age (median): 46.7 years Sex: male (42%) Setting: community setting	Framework analysis and thematic analysis	Intervention: Electronic monitoring device (EMD) Component: Mobile phone app	Data inaccuracy leads to user disengagement Intervention could facilitate timely support by healthcare team Real-time adherence data enhances patient knowledge of their adherence and boosts confidence to discuss concerns with healthcare provider Digital technology should be embedded within the inhaler Adherence feedback to be co-ordinated by primary healthcare Clinician feedback to be given as part of the asthma review process To integrate inhalation data with other data such as activity levels, physiological data, and environmental factors
Van Emmenis et al. [43] United kingdom	To explore patients’ and HCP views on (1) non-adherence to hypertension medication and (2) a complex intervention designed to support medication adherence	Study design: Qualitative study Data collection method: Semi structured interviews and focus groups	Sample size: n = 31 Patient group: hypertensive patients Type 2 diabetes Age (median): 67 years Sex: male (60%) Setting: primary care practice	Constant comparison Analysis and Perceptions and Practicalities (PAPA) approach framework	Intervention: Digital intervention (DI) Very brief face to face intervention (VBI) Component: Reminders, text messages, adherence feedback, sensing technology and a VBI protocol	Adherence reminders and feedback messages are acceptable Sensing technology beneficial but participants expressed concerns about data privacy
Smith et al. [44] United States	To examine patients’ views on a mobile medication adherence application for enhancing adherence to opioid use disorder treatment	Study design: Quantitative descriptive study Data collection Method: Survey including closed and open- ended questions	Sample size: n = 20 Patient group: People diagnosed with opioid use disorder and prescribed with opioid substitution therapy Age: 18 years or older Sex: male (35%) Setting: addiction medicine clinics	Descriptive statistics	Intervention: Mobile medication adherence reminder app Component: Bluetooth-enabled pill bottle cap Text messages Automated phone calls	Participants suggested integration of personalised reminders, motivational features, web-based coaching, reward based incentives and behaviour trackers to enhance adherence

*ACS* acute coronary syndrome, *App* application, *ART* antiretroviral therapy, *DAT* digital adherence tools, *EIDM* electronic medication inhalation device, *EMD* electronic monitoring device, *HCP* healthcare provider, *HTN* hypertension, *IVR* interactive voice response, *MED* MEssaging for diabetes, *MSI* mass spectrometry imaging, *PCI* percutaneous intervention, *SMS* short messaging service, *T2DM* type 2 diabetes mellitus, *VBI* very brief face to face intervention

### Quality assessment

While the MMAT does not produce an overall score for quality, the colour-coded responses indicated that, beyond the initial screening questions, most studies met the quality criteria (“Yes”) or had insufficient information to determine quality (“Can’t tell”). No studies were excluded based on the quality assessment (Table 3).

**Table 3:**
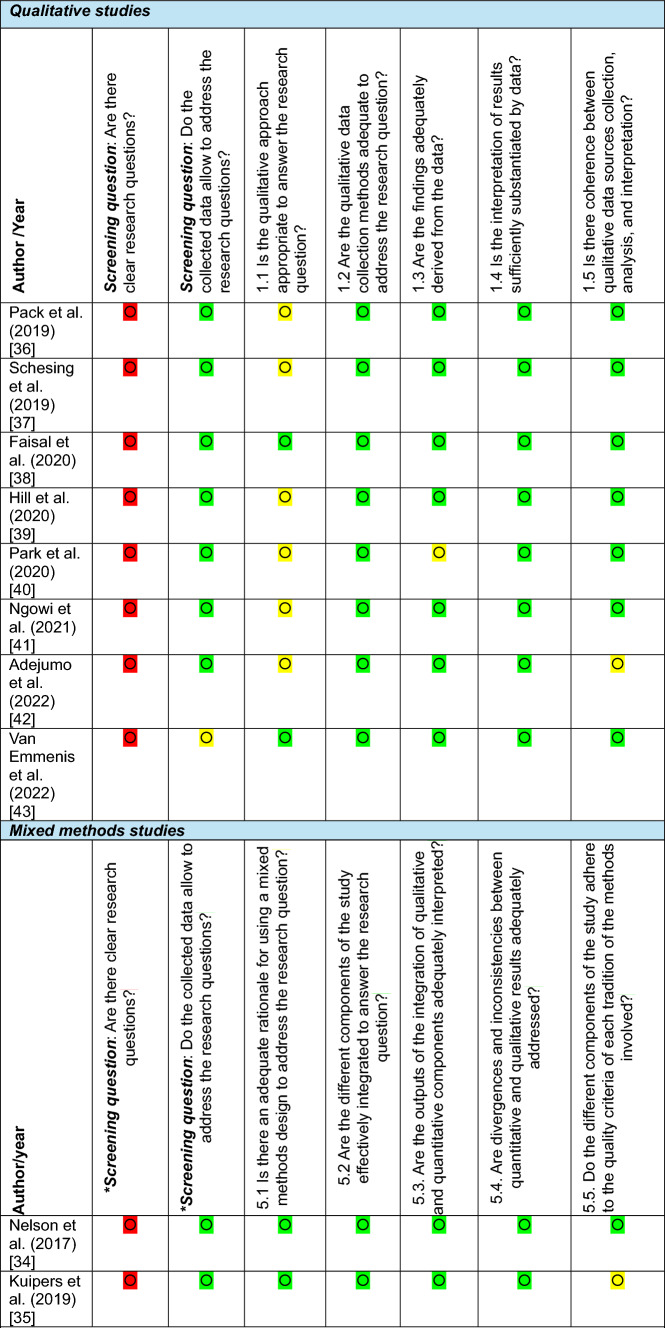

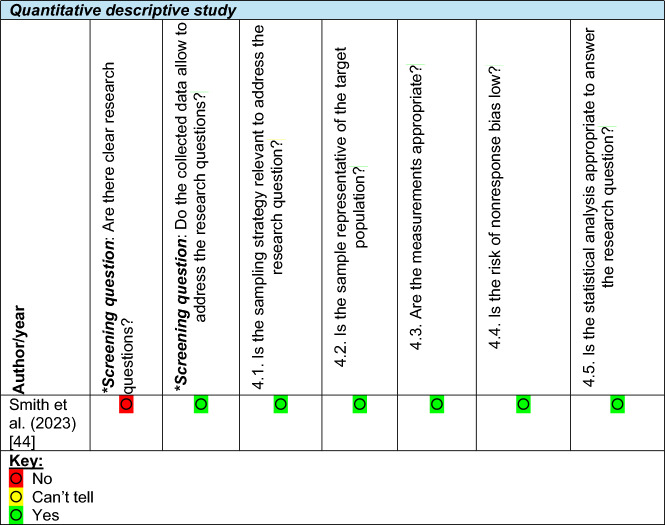
Quality assessment of included studies using the mixed methods appraisal tool (MMAT)

### Data analysis

Intervention components across studies included mobile apps, SMS (short messaging service) texts, interactive voice response (IVR) sensing technology such as the GPS (Global positioning system) and electronic monitoring of adherence. Three overarching analytical themes were developed and categorised into sub-themes. These were ‘balancing support and autonomy in feedback interventions’, ‘maintaining patient-provider relationship’ and ‘enhancing engagement through tailored design’. Examples of participant quotes relevant to each theme have been presented in Table 3.

### Balancing support and autonomy in feedback interventions

#### Perceived value and acceptability of adherence feedback interventions

Overall, feedback interventions were perceived as valuable in enhancing participants' awareness of their medication-taking behaviours. Simple to use interventions were acceptable if they provided text message reminders, motivational, emotional or practical support [34, 35, 40–42, 44]. Text message reminders that provided daily or weekly reminders, motivational messages or advice on when to seek expert healthcare advice had mixed reviews, with some participants finding them valuable and others perceiving them as bothersome. This highlights the need for individual assessments to inform the selection of appropriate adherence interventions that support medication optimisation across diverse patient groups or for interventions to be customisable for or by the patient.

Data presentation was considered essential for supporting understanding of adherence patterns. Some interventions provided feedback verbally, via automated response or graphical features [34–37, 40, 42–44]. Although there were mixed views, participants found graphical adherence data presentations, mostly bar graphs, acceptable [36, 41, 43]. Some participants also valued the use of schematic colour codes to simplify the data and expressed their preference for this approach [40]. These methods were recognised for their ability to enhance understanding of medication adherence patterns to improve behaviour.

#### Navigating trust, data security and technical reliability

Participants expressed concern with some intervention features. For example, some wanted to have control over who could gain access to their data when using digital technologies involving apps and monitoring systems. [43]. They sought reassurance that their health information would be securely managed and protected from unsolicited use [35, 40, 42, 43] and suggested that data should be handled by regulated healthcare organisations as they believed this would enhance data integrity and security [43]. Intervention product size was also of concern for participants, especially if the device was obtrusive [38, 41]. This indicated an emphasis on privacy in medicines management. Additionally, some participants reported that technical problems had led to inaccurate or delayed feedback raising concerns around intervention functionality [34, 35, 38]. These challenges highlighted barriers to intervention acceptability, with some participants expressing reluctance to engage with interventions beyond the study [42]. Others, however, indicated that addressing technical issues could readily enhance user engagement [35].

### Maintaining patient-provider relationship

Objective feedback on adherence behaviour was delivered remotely [34, 42, 44] or directly by the intervention team [35–39, 41, 43]. Nonetheless, some participants expressed their preference to maintain contact with healthcare providers (HCPs) to be reassured of expert monitoring. Maintaining good relationships with healthcare providers when using interventions was perceived to enhance shared decision-making and encourage relationship-building through empathy and conversations that were non-judgemental [37, 39, 43, 44]. Additionally, access to adherence information and an enhanced sense of autonomy over one's health were identified as beneficial outcomes of such relationships. Some participants highlighted the need for interventions that facilitated shared access to real-time medication adherence data between patients and providers, enabling collaborative problem-solving [40]. Although receiving objective adherence feedback was well accepted [34, 36, 39, 42], there were concerns about the negative impact it could have on patient-provider relationships [37, 39]. This posed a potential barrier to using objective data for assessing adherence, but others suggested that a compassionate approach by providers would foster trust [37].

### Enhancing engagement through tailored design

#### User needs determine intervention engagement

Studies highlighted the importance of person-centred interventions [34, 35, 38, 40, 41, 43, 44]. Age or health literacy levels, ease of use and personalised messages targeting specific needs including polypharmacy were considered essential for appropriate intervention selection and user engagement [35, 38, 43]. Those with recent diagnoses of diabetes found text messages and app interventions more useful than those with longer-standing diagnoses, because it supported routine-building. Others emphasised the importance of interventions that address all prescribed medications, rather than focusing on a single medication, particularly for individuals managing multiple conditions [35, 38, 43]. Such preferences underlined the need for healthcare providers to identify individual needs prior to selecting adherence support intervention [34, 35, 38].

#### Suggestions for improvement

Participants suggested incorporating audio and visual cues [38, 42], real-time longitudinal data or colour coded calendars to prompt adherence and identify inconsistencies in adherence patterns [36]. They also preferred interventions tailored to user characteristics, such as age or length of diagnosis over generic approaches [34]. Some participants expressed preference for simple-to-use interventions [35, 38] with personalised features [34, 40, 41, 44]. Others believed that a common adherence feedback interface would facilitate joint decision-making with healthcare providers [40]. Furthermore, integration of physiological, environmental and activity related data with adherence data proved appealing [42]. These suggestions could inform the development of tailored interventions that are aligned with user preferences and incorporate features that facilitate effective self-management and enhance engagement (Table 4).

**Table 4:** Participant quotes from included studies

Theme	Context	Quotes
Balancing support and autonomy in feedback interventions	Participants’ views on daily or weekly text message reminders, motivational and emotional support	“When those text messages came, I would be getting ready to take my bedtime dose, so it helped me remember to take it, if I was already sleepy.” (Participant—Nelson et al. 2021) [34] “The messages were very helpful, informative, and encouraging. It felt like I had my own personal cheerleader.” (Participant—Nelson et al.2021) [34] “It’s kind of, like, ‘okay little boy, and are you gonna do this today?’ It’s annoying, treating me like a little child.” (Participant—Park et al. 2020) [40] “The texts were not helpful to me because I’ve had diabetes for so long, but they could be very helpful to those just diagnosed.” (Participant—Nelson et al. 2021). [34]
	Participants’ views on adherence data presentation (graphical presentation and colour coded schemes)	“I want to see what my health looks like in the long-term, not just on my day-to-day of just remembering to take my medications… If you have a graph or chart, you know, it’s in your face kind of thing.” (Participant—Park et al. 2020) [40] “It's cool that it can tell you that far back... I think it would give you a better idea of how good you do at taking your medicine and help you keep it in check.” (Participant—Pack et al. 2019) [36] “If you get a little happy face pillow or something that indicated good adherence and good practices, good viral load management, or just an ongoing awareness of the fact that you’re HIV-positive and you’re sticking to your prescribed behaviours…” (Participant—Hill et al. 2021) [39] “I guess it’s pretty basic, where it’s almost like green-go, and red-stop, or red-danger.” (Participant—Park et al. 2020) [40]
	Concerns with data security, intervention practicalities and technical challenges	“I think it’d be more reassuring to know it was a medical body behind it or a university body behind it; it gives it some substance and credibility.” (Participant—Van Emmenis et al. 2022) [43] “Like I’m going out for lunch with my women’s group. I can’t take this flying saucer with me. It’s not going to fit in my…the purse that I want to bring. So, … I’m probably gonna miss the dose by being out.” (Participant—Faisal et al. 2020) [38] “Until now, the app missed three inhalation registrations in the morning. I clicked twice within a minute, but only one [inhalation] was registered”. (Participant -Kuipers et al. 2019) [35]
Maintaining patient-provider relationship	Participants’ views and suggestions on relationship building	“It would be useful for a physician’s office to keep track of you because the biggest problem is non-compliance…it helps to know the doctor will be checking.” (Participant—Park et al. 2020) [40] “I think it might be worthwhile for the doctor to sit down, and say,.. we want you to know that nobody is perfect, and we just want to help you get healthier.” (Participant—Hill et al. 2021) [39] “You want him (the doctor) to tell you what's right but in a compassionate way. Nobody likes to be yelled at…” (Participant—Schesing et al. 2019) [37]
Enhancing engagement through tailored design	Participants' needs and preferences	“I went from basically not really taking any medication to now I take about nine….so I’m kind of learning a new routine. Text messaging could help with the routine.” (Participant -Park et al. 2020) [40] “I wanted more variation in messages … I needed more encouragement rather than repeating the same facts.” (Participant—Nelson et al. 2021) [34] “Some people are on medication once a day, twice a day, three, four. Could the app be tailor-made for the individuals and remind us accordingly?” (Participant—Van Emmenis et al. 2022) [43]

## Discussion

### Statement of key findings

As far as we are aware, this review is the first to synthesise data on patients’ perspectives on medication adherence feedback interventions. Findings identified a variety of adherence feedback interventions and reported on participants’ acceptability and preferences. Interventions that were simple and easy to use and facilitated effective communication with healthcare providers were of importance to participants and suggestions to personalise interventions were key elements to enhance intervention engagement.

### Interpretation

Adherence feedback interventions were deemed acceptable and easy to use and included eHealth technologies such as mobile phones, mobile apps, text messaging services and electronic monitoring. Data security and technical challenges were barriers to intervention acceptability. This is consistent with the literature that users value transparency and control over who has access to their personal health data [45–47]. Although not always a concern for users [48], our findings suggest that addressing these barriers would enhance intervention engagement.

Previous eHealth adherence intervention studies have included similar intervention components as identified from this review [14, 49]. While these studies focused on intervention effectiveness, our review adds to this by synthesising evidence on users’ perceptions. Findings suggest interventions were acceptable because they instilled behavioural changes when used as reminders, adherence monitoring and motivational tools. This may be attributed to the integration of behaviour change techniques that align with established theoretical frameworks such as the Theoretical Domains Framework (TDF)[50]. Based on these findings, the key domains from the TDF which any underpinning behavioural theory should address include behavioural regulation, knowledge, memory, motivation and goals [50]. The importance placed on the input of the healthcare provider also suggests that patient-professional role and identity should be addressed.

Despite the growing adoption of EHR for obtaining actionable information from patient healthcare data, this area remains unexplored in the current literature. Literature confirms this paucity and posits that EHR could better support those living with chronic conditions and polypharmacy by empowering them to manage their health [23, 51]. Advantages including objective data, cost-effectiveness and the automatic generation of medication adherence histories enhance the scalability of EHR interventions [23]. Further research should therefore explore how best to utilise EHR to optimise adherence.

Similar to other study findings, our review identified effective communication between patients and providers as a key factor in supporting adherence. A rapid review of five qualitative studies reported that good communication enhanced shared decision-making and influenced patients’ adherence beliefs [52], confirming our findings. Furthermore, our findings proposed that involving HCPs through a shared adherence data interface can strengthen communication and improve adherence behaviour. This suggests that feedback interventions are not replacement tools but rather adjuncts that support person-centred adherence. Future research should consider how tailored HCP support can be integrated into feedback interventions.

Real-time longitudinal data presented with graphical features were considered useful although could present a challenge for individuals with lower health literacy. This finding aligned with studies that found visual data to be simple yet effective communication tools compared to verbal or textual formats [53]. The use of colour codes in visual displays were also found useful in highlighting adherence issues and prompting quick action to address them. As such, incorporating these features in feedback interventions could enhance user understanding of adherence patterns and improve behaviour.

Although eHealth feedback interventions were deemed acceptable, participants offered suggestions for improvement. Most notably, personalised interventions were vital to their success. A systematic review of 35 qualitative and mixed-methods studies emphasised the need for personalised features in eHealth interventions [54]. Though not specific to medication adherence, the findings reinforce the importance of addressing user needs and involving them in the design process, as recommended by complex intervention development guidelines [55]. However, user involvement in intervention design was not reported in the reviewed studies, aligning with a systematic review of 31 studies that identified significant gaps in patient and public participation in healthcare intervention development [56]. Our findings indicate that tailored interventions are essential, and that participants play a key role in their design and development. While challenging [57], literature consistently supports involving patients and the public in healthcare intervention development [58, 59], an approach future research on adherence feedback interventions should adopt.

### Strengths and weaknesses

Our review used robust methodology to search, identify and synthesise qualitative evidence. This review is the first to synthesise qualitative evidence on patients’ perceptions on medication adherence feedback interventions. The multidisciplinary composition of the authors (nursing, psychology, and pharmacy professionals) may have introduced interpretive biases regarding usability and adaptability of feedback interventions for long-term medication management in chronic disease. Another limitation was the predominant representation of studies conducted in high-resource settings, which constrains the generalisability of findings to low-resource contexts. Future research should examine how these findings apply across diverse healthcare systems.

### Further research

Further research should incorporate robust design methodologies that involve users in adherence feedback intervention development. This would help identify user needs and develop appropriate support solutions to improve adherence and intervention engagement, as highlighted by the review. eHealth monitoring and feedback interventions are not always cost-effective or successfully scalable [21]. Therefore, further research should adopt alternative approaches, such as leveraging electronic health record data, to enhance cost-effectiveness and scalability, making interventions more accessible to wider populations. Future research should also develop and test feedback interventions that provide real-time objective data through visual presentations. This would enhance patient-provider communication, improve understanding of adherence patterns, and support shared decision-making. Additionally, intervention developers should incorporate relevant health behaviour change theories into intervention designs to improve adherence outcomes, while ensuring transparency for replication and further refinement [55, 60, 61].

## Conclusion

In conclusion, technology-mediated feedback interventions are effective when easy to use, though privacy concerns and technical issues can hinder engagement. Their success relies on user acceptability, HCP involvement, and incorporating person-centred design. Integrating behaviour change theories, EHR systems, and visual formats to communicate adherence data can enhance support and improve adherence. Future research should address barriers, involve users in intervention design, and leverage EHR medication adherence data to empower users, foster engagement, and optimise adherence.

## Supplementary Information

Below is the link to the electronic supplementary material.

Supplementary file1 (DOCX 2444 KB)

## Data Availability

No datasets were generated or analysed during the current study.
