# Supplementary material for: Patients’ perspectives on medication adherence feedback interventions for managing long-term medications: a systematic review of qualitative evidence
Source: Int J Clin Pharm. 2025 Jul 16;48(1):1–16. doi: 10.1007/s11096-025-01958-4 (PMC12823734; doi:10.1007/s11096-025-01958-4)

DATABASE SEARCH TERMS AND STRATEGY (Updated February 2023 – February 2025)

**CINAHL database Search (Date:13/02/2023)**


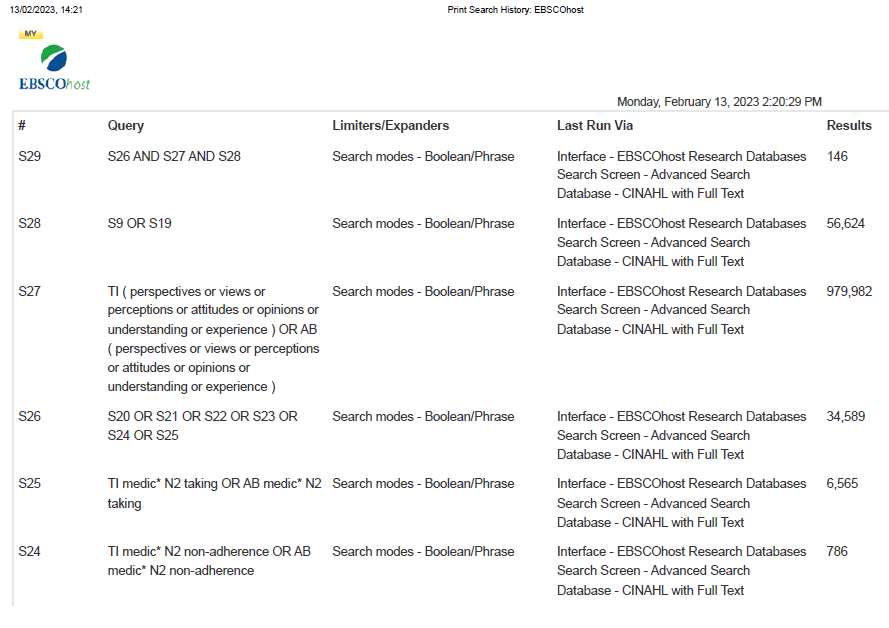


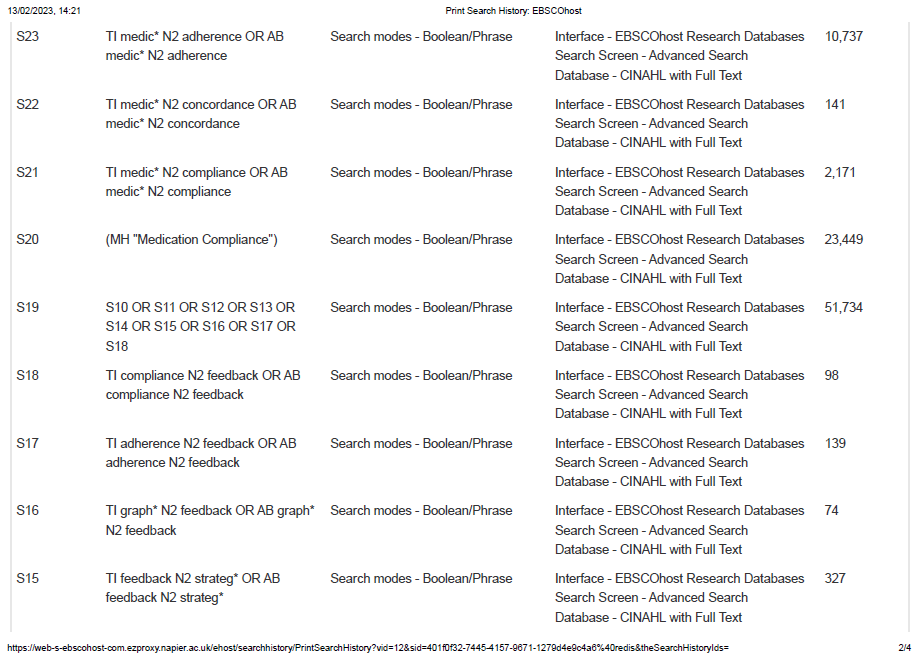


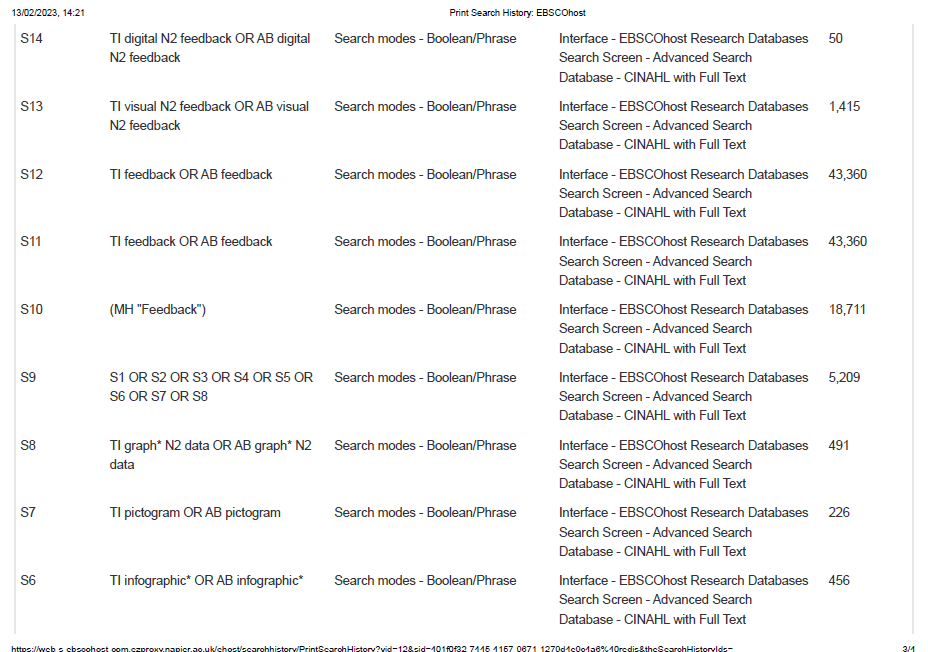


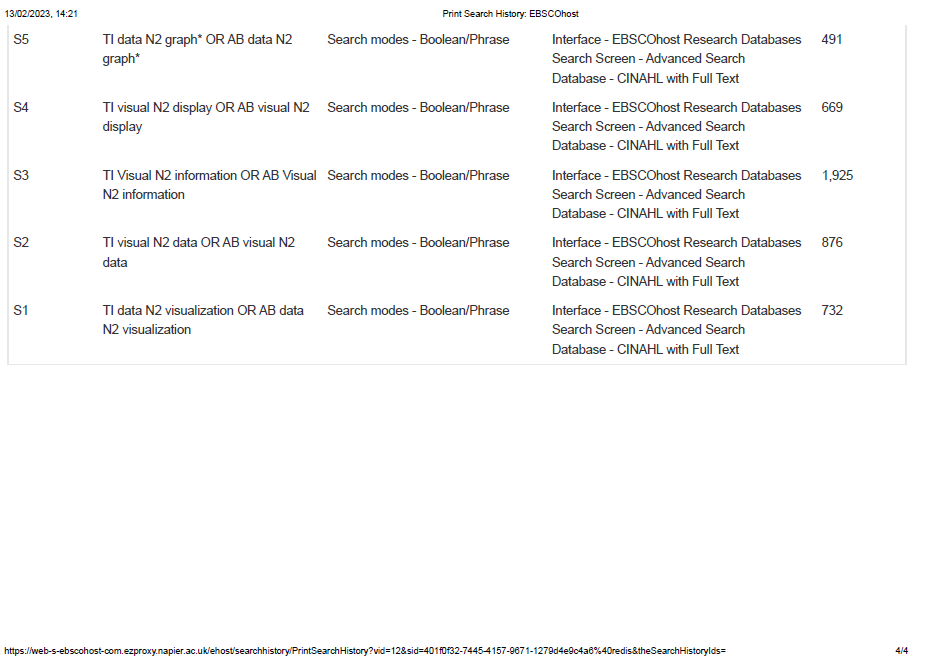


**EMBASE database Search (Date:13/02/2023)**

Embase: 1974 to 2023 February

1 exp data visualization/ 2419

2 data visualization.tw. 2528

3 visual data.tw. 891

4 visual information.tw. 11592

5 visual display.tw. 2076

6 data graph*.tw. 306

7 infographic*.tw. 1207

8 pictogram.tw. 484

9 graph data.tw. 219

10 1 or 2 or 3 or 4 or 5 or 6 or 7 or 8 or 9 20729

11 feedback.tw. 218499

12 visual feedback.tw. 5932

13 digital feedback.tw. 81

14 feedback strateg*.tw. 359

15 graph* feedback.tw. 94

16 adherence feedback.tw. 65

17 compliance feedback.tw. 26

18 11 or 12 or 13 or 14 or 15 or 16 or 17 218499

19 exp medication compliance/ 43391

20 medic* compliance.tw. 3707

21 medic* concordance.tw. 60

22 medic* adherence.tw. 22732

23 medic* non-adherence.tw. 1955

24 medic* taking.tw. 2076

25 19 or 20 or 21 or 22 or 23 or 24 55671

26 (perspectives or views or perceptions or attitudes or opinions or understanding or experience).tw. 2866148

27 10 or 18 237994

28 25 and 26 and 27 294

**MEDLINE database Search (Date:13/02/2023)**

**
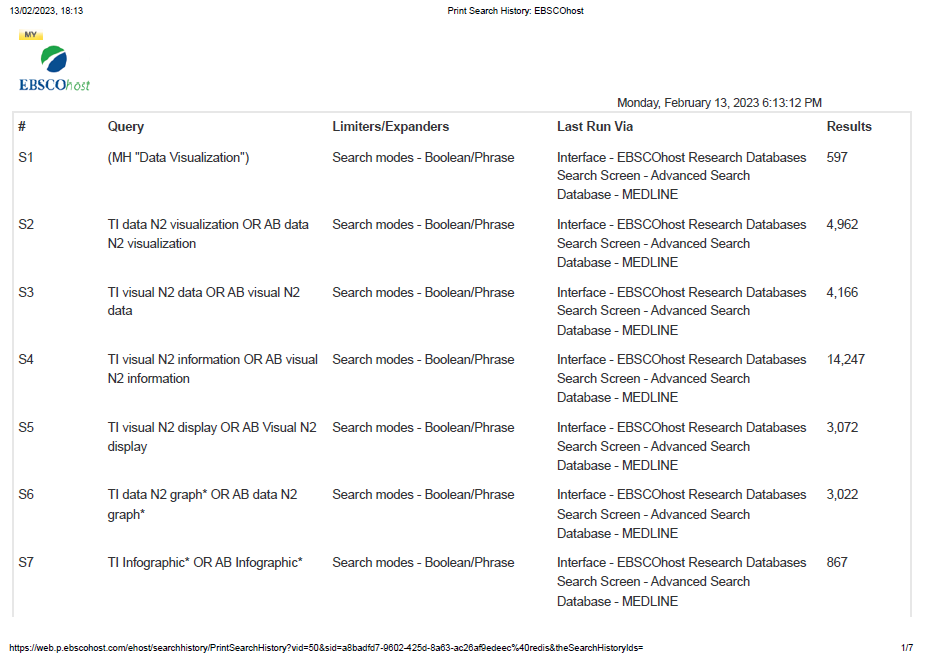
**

**
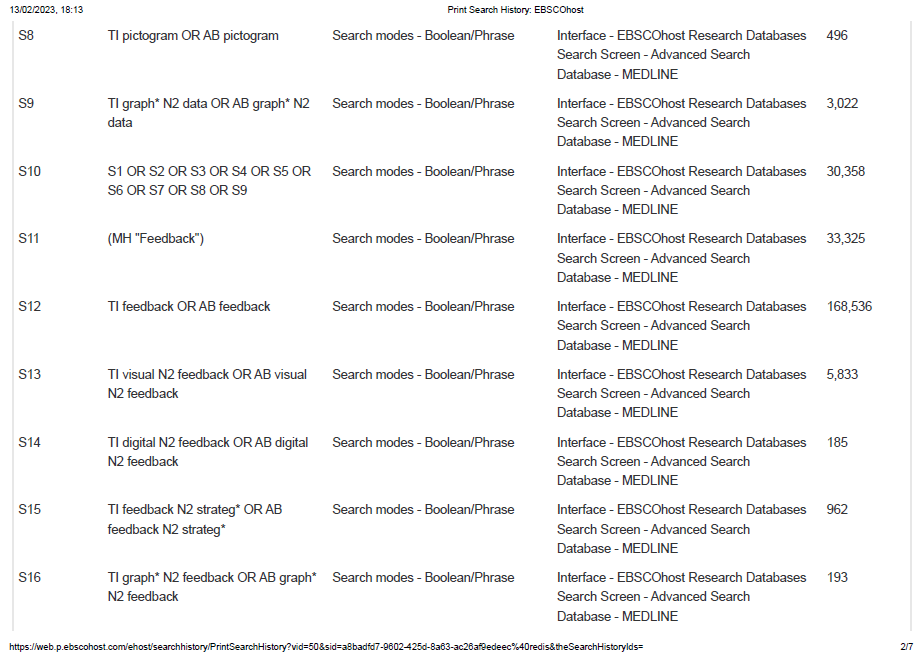
**


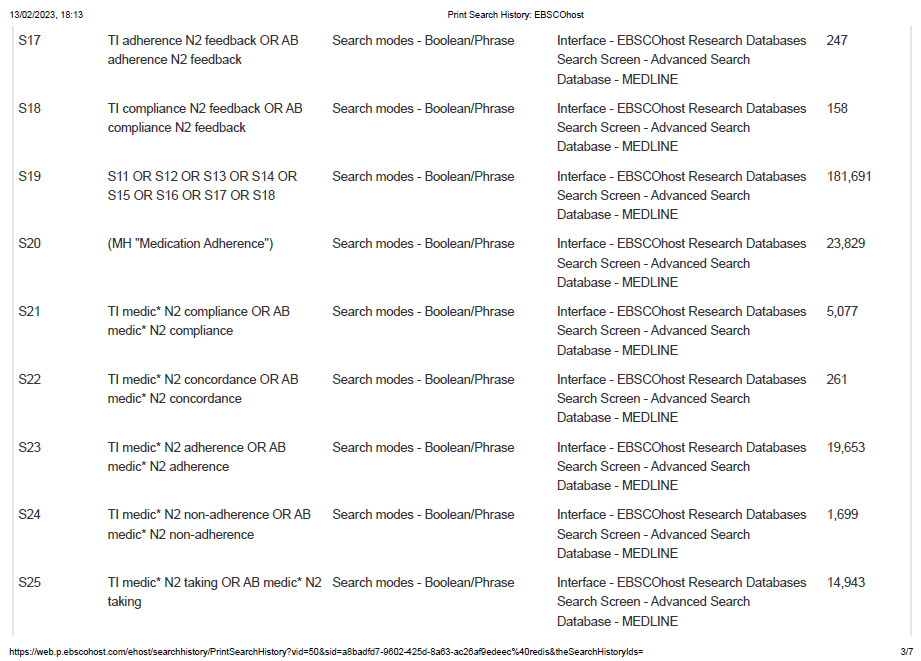


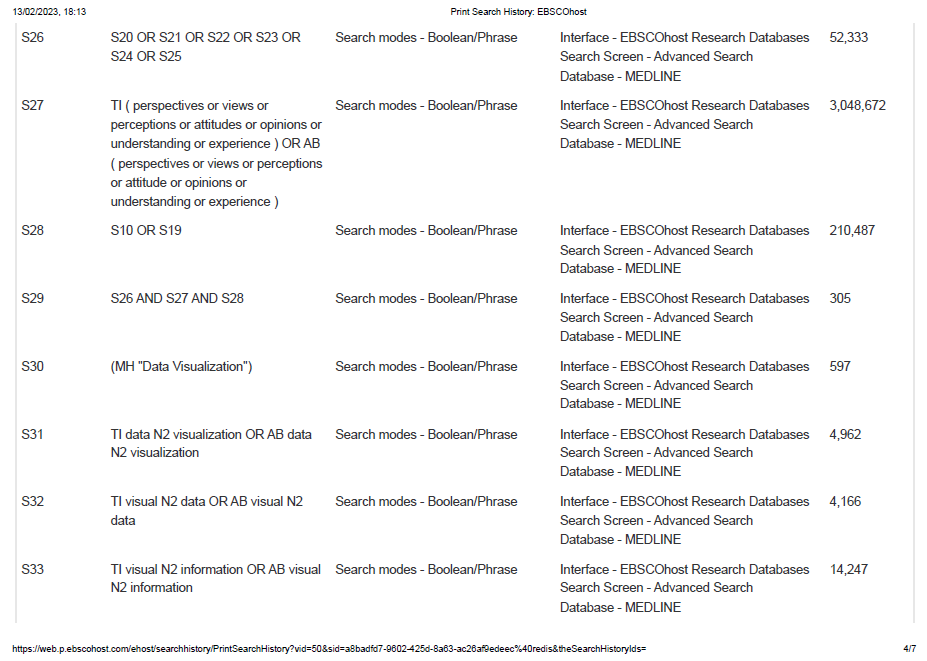


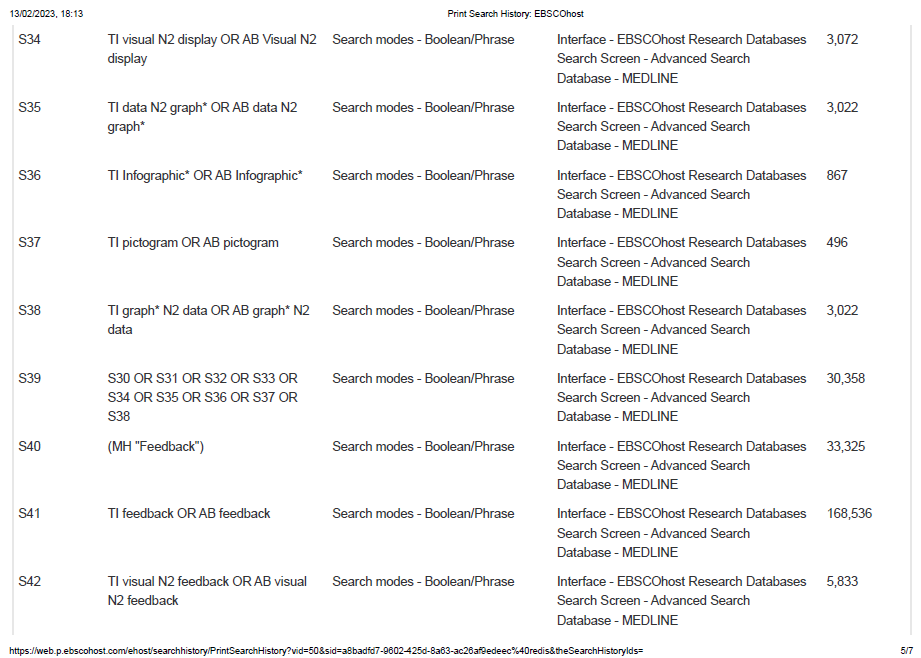


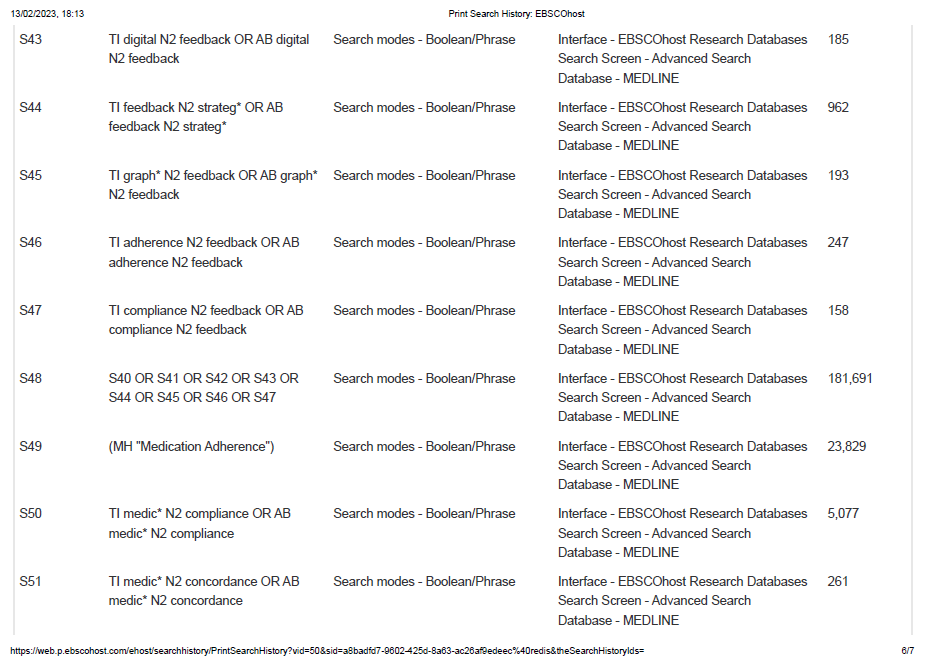


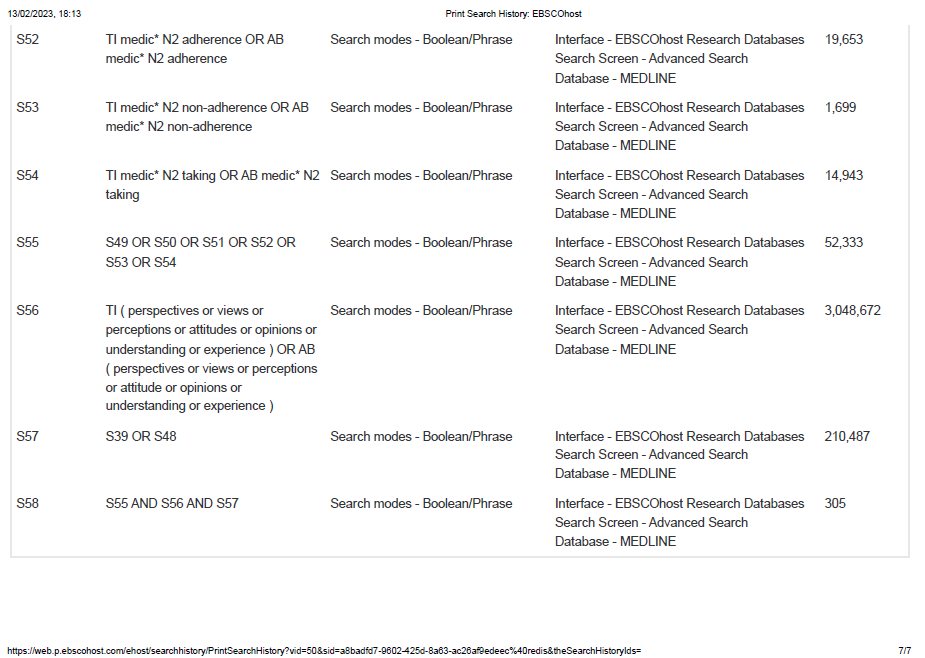


**APA PsycInfo database search (Date: 13/02/2023)**

| **#** | **Query** | **Limiters/Expanders** | **Results** | **Action** |
| --- | --- | --- | --- | --- |
| S1 | (MH "Data Visualization") | Search modes - Boolean/Phrase | 0 | [Edit](javascript:__doPostBack('ctl00$ctl00$MainContentArea$MainContentArea$editControl$printHistory$HistoryRepeater$ctl00$linkEditSearch',''))S1 |
| S2 | TI data N2 visualization OR AB data N2 visualization | Search modes - Boolean/Phrase | 707 | [Edit](javascript:__doPostBack('ctl00$ctl00$MainContentArea$MainContentArea$editControl$printHistory$HistoryRepeater$ctl01$linkEditSearch',''))S2 |
| S3 | TI visual N2 data OR AB visual N2 data | Search modes - Boolean/Phrase | 1,879 | [Edit](javascript:__doPostBack('ctl00$ctl00$MainContentArea$MainContentArea$editControl$printHistory$HistoryRepeater$ctl02$linkEditSearch',''))S3 |
| S4 | TI visual N2 information OR AB visual N2 information | Search modes - Boolean/Phrase | 11,707 | [Edit](javascript:__doPostBack('ctl00$ctl00$MainContentArea$MainContentArea$editControl$printHistory$HistoryRepeater$ctl03$linkEditSearch',''))S4 |
| S5 | TI visual N2 display OR AB Visual N2 display | Search modes - Boolean/Phrase | 2,758 | [Edit](javascript:__doPostBack('ctl00$ctl00$MainContentArea$MainContentArea$editControl$printHistory$HistoryRepeater$ctl04$linkEditSearch',''))S5 |
| S6 | TI data N2 graph* OR AB data N2 graph* | Search modes - Boolean/Phrase | 1,040 | [Edit](javascript:__doPostBack('ctl00$ctl00$MainContentArea$MainContentArea$editControl$printHistory$HistoryRepeater$ctl05$linkEditSearch',''))S6 |
| S7 | TI Infographic* OR AB Infographic* | Search modes - Boolean/Phrase | 146 | [Edit](javascript:__doPostBack('ctl00$ctl00$MainContentArea$MainContentArea$editControl$printHistory$HistoryRepeater$ctl06$linkEditSearch',''))S7 |
| S8 | TI pictogram OR AB pictogram | Search modes - Boolean/Phrase | 255 | [Edit](javascript:__doPostBack('ctl00$ctl00$MainContentArea$MainContentArea$editControl$printHistory$HistoryRepeater$ctl07$linkEditSearch',''))S8 |
| S9 | TI graph* N2 data OR AB graph* N2 data | Search modes - Boolean/Phrase | 1,040 | [Edit](javascript:__doPostBack('ctl00$ctl00$MainContentArea$MainContentArea$editControl$printHistory$HistoryRepeater$ctl08$linkEditSearch',''))S9 |
| S10 | S1 OR S2 OR S3 OR S4 OR S5 OR S6 OR S7 OR S8 OR S9 | Search modes - Boolean/Phrase | 17,989 | [Edit](javascript:__doPostBack('ctl00$ctl00$MainContentArea$MainContentArea$editControl$printHistory$HistoryRepeater$ctl09$linkEditSearch',''))S10 |
| S11 | (MH "Feedback") | Search modes - Boolean/Phrase | 4 | [Edit](javascript:__doPostBack('ctl00$ctl00$MainContentArea$MainContentArea$editControl$printHistory$HistoryRepeater$ctl10$linkEditSearch',''))S11 |
| S12 | TI feedback OR AB feedback | Search modes - Boolean/Phrase | 75,761 | [Edit](javascript:__doPostBack('ctl00$ctl00$MainContentArea$MainContentArea$editControl$printHistory$HistoryRepeater$ctl11$linkEditSearch',''))S12 |
| S13 | TI visual N2 feedback OR AB visual N2 feedback | Search modes - Boolean/Phrase | 3,238 | [Edit](javascript:__doPostBack('ctl00$ctl00$MainContentArea$MainContentArea$editControl$printHistory$HistoryRepeater$ctl12$linkEditSearch',''))S13 |
| S14 | TI digital N2 feedback OR AB digital N2 feedback | Search modes - Boolean/Phrase | 97 | [Edit](javascript:__doPostBack('ctl00$ctl00$MainContentArea$MainContentArea$editControl$printHistory$HistoryRepeater$ctl13$linkEditSearch',''))S14 |
| S15 | TI feedback N2 strateg* OR AB feedback N2 strateg* | Search modes - Boolean/Phrase | 693 | [Edit](javascript:__doPostBack('ctl00$ctl00$MainContentArea$MainContentArea$editControl$printHistory$HistoryRepeater$ctl14$linkEditSearch',''))S15 |
| S16 | TI graph* N2 feedback OR AB graph* N2 feedback | Search modes - Boolean/Phrase | 221 | [Edit](javascript:__doPostBack('ctl00$ctl00$MainContentArea$MainContentArea$editControl$printHistory$HistoryRepeater$ctl15$linkEditSearch',''))S16 |
| S17 | TI adherence N2 feedback OR AB adherence N2 feedback | Search modes - Boolean/Phrase | 74 | [Edit](javascript:__doPostBack('ctl00$ctl00$MainContentArea$MainContentArea$editControl$printHistory$HistoryRepeater$ctl16$linkEditSearch',''))S17 |
| S18 | TI compliance N2 feedback OR AB compliance N2 feedback | Search modes - Boolean/Phrase | 42 | [Edit](javascript:__doPostBack('ctl00$ctl00$MainContentArea$MainContentArea$editControl$printHistory$HistoryRepeater$ctl17$linkEditSearch',''))S18 |
| S19 | S11 OR S12 OR S13 OR S14 OR S15 OR S16 OR S17 OR S18 | Search modes - Boolean/Phrase | 75,761 | [Edit](javascript:__doPostBack('ctl00$ctl00$MainContentArea$MainContentArea$editControl$printHistory$HistoryRepeater$ctl18$linkEditSearch',''))S19 |
| S20 | (MH "Medication Adherence") | Search modes - Boolean/Phrase | 0 | [Edit](javascript:__doPostBack('ctl00$ctl00$MainContentArea$MainContentArea$editControl$printHistory$HistoryRepeater$ctl19$linkEditSearch',''))S20 |
| S21 | TI medic* N2 compliance OR AB medic* N2 compliance | Search modes - Boolean/Phrase | 1,878 | [Edit](javascript:__doPostBack('ctl00$ctl00$MainContentArea$MainContentArea$editControl$printHistory$HistoryRepeater$ctl20$linkEditSearch',''))S21 |
| S22 | TI medic* N2 concordance OR AB medic* N2 concordance | Search modes - Boolean/Phrase | 72 | [Edit](javascript:__doPostBack('ctl00$ctl00$MainContentArea$MainContentArea$editControl$printHistory$HistoryRepeater$ctl21$linkEditSearch',''))S22 |
| S23 | TI medic* N2 adherence OR AB medic* N2 adherence | Search modes - Boolean/Phrase | 6,934 | [Edit](javascript:__doPostBack('ctl00$ctl00$MainContentArea$MainContentArea$editControl$printHistory$HistoryRepeater$ctl22$linkEditSearch',''))S23 |
| S24 | TI medic* N2 non-adherence OR AB medic* N2 non-adherence | Search modes - Boolean/Phrase | 601 | [Edit](javascript:__doPostBack('ctl00$ctl00$MainContentArea$MainContentArea$editControl$printHistory$HistoryRepeater$ctl23$linkEditSearch',''))S24 |
| S25 | TI medic* N2 taking OR AB medic* N2 taking | Search modes - Boolean/Phrase | 4,290 | [Edit](javascript:__doPostBack('ctl00$ctl00$MainContentArea$MainContentArea$editControl$printHistory$HistoryRepeater$ctl24$linkEditSearch',''))S25 |
| S26 | S20 OR S21 OR S22 OR S23 OR S24 OR S25 | Search modes - Boolean/Phrase | 12,371 | [Edit](javascript:__doPostBack('ctl00$ctl00$MainContentArea$MainContentArea$editControl$printHistory$HistoryRepeater$ctl25$linkEditSearch',''))S26 |
| S27 | TI ( perspectives or views or perceptions or attitudes or opinions or understanding or experience ) OR AB ( perspectives or views or perceptions or attitude or opinions or understanding or experience ) | Search modes - Boolean/Phrase | 1,694,580 | [Edit](javascript:__doPostBack('ctl00$ctl00$MainContentArea$MainContentArea$editControl$printHistory$HistoryRepeater$ctl26$linkEditSearch',''))S27 |
| S28 | S10 OR S19 | Search modes - Boolean/Phrase | 92,838 | [Edit](javascript:__doPostBack('ctl00$ctl00$MainContentArea$MainContentArea$editControl$printHistory$HistoryRepeater$ctl27$linkEditSearch',''))S28 |
| S29 | S26 AND S27 AND S28 | Search modes - Boolean/Phrase | 61 | [Edit](javascript:__doPostBack('ctl00$ctl00$MainContentArea$MainContentArea$editControl$printHistory$HistoryRepeater$ctl28$linkEditSearch',''))S29 |
| S30 | (MH "Data Visualization") | Search modes - Boolean/Phrase | 0 | [Edit](javascript:__doPostBack('ctl00$ctl00$MainContentArea$MainContentArea$editControl$printHistory$HistoryRepeater$ctl29$linkEditSearch',''))S30 |
| S31 | TI data N2 visualization OR AB data N2 visualization | Search modes - Boolean/Phrase | 707 | [Edit](javascript:__doPostBack('ctl00$ctl00$MainContentArea$MainContentArea$editControl$printHistory$HistoryRepeater$ctl30$linkEditSearch',''))S31 |
| S32 | TI visual N2 data OR AB visual N2 data | Search modes - Boolean/Phrase | 1,879 | [Edit](javascript:__doPostBack('ctl00$ctl00$MainContentArea$MainContentArea$editControl$printHistory$HistoryRepeater$ctl31$linkEditSearch',''))S32 |
| S33 | TI visual N2 information OR AB visual N2 information | Search modes - Boolean/Phrase | 11,707 | [Edit](javascript:__doPostBack('ctl00$ctl00$MainContentArea$MainContentArea$editControl$printHistory$HistoryRepeater$ctl32$linkEditSearch',''))S33 |
| S34 | TI visual N2 display OR AB Visual N2 display | Search modes - Boolean/Phrase | 2,758 | [Edit](javascript:__doPostBack('ctl00$ctl00$MainContentArea$MainContentArea$editControl$printHistory$HistoryRepeater$ctl33$linkEditSearch',''))S34 |
| S35 | TI data N2 graph* OR AB data N2 graph* | Search modes - Boolean/Phrase | 1,040 | [Edit](javascript:__doPostBack('ctl00$ctl00$MainContentArea$MainContentArea$editControl$printHistory$HistoryRepeater$ctl34$linkEditSearch',''))S35 |
| S36 | TI Infographic* OR AB Infographic* | Search modes - Boolean/Phrase | 146 | [Edit](javascript:__doPostBack('ctl00$ctl00$MainContentArea$MainContentArea$editControl$printHistory$HistoryRepeater$ctl35$linkEditSearch',''))S36 |
| S37 | TI pictogram OR AB pictogram | Search modes - Boolean/Phrase | 255 | [Edit](javascript:__doPostBack('ctl00$ctl00$MainContentArea$MainContentArea$editControl$printHistory$HistoryRepeater$ctl36$linkEditSearch',''))S37 |
| S38 | TI graph* N2 data OR AB graph* N2 data | Search modes - Boolean/Phrase | 1,040 | [Edit](javascript:__doPostBack('ctl00$ctl00$MainContentArea$MainContentArea$editControl$printHistory$HistoryRepeater$ctl37$linkEditSearch',''))S38 |
| S39 | S30 OR S31 OR S32 OR S33 OR S34 OR S35 OR S36 OR S37 OR S38 | Search modes - Boolean/Phrase | 17,989 | [Edit](javascript:__doPostBack('ctl00$ctl00$MainContentArea$MainContentArea$editControl$printHistory$HistoryRepeater$ctl38$linkEditSearch',''))S39 |
| S40 | (MH "Feedback") | Search modes - Boolean/Phrase | 4 | [Edit](javascript:__doPostBack('ctl00$ctl00$MainContentArea$MainContentArea$editControl$printHistory$HistoryRepeater$ctl39$linkEditSearch',''))S40 |
| S41 | TI feedback OR AB feedback | Search modes - Boolean/Phrase | 75,761 | [Edit](javascript:__doPostBack('ctl00$ctl00$MainContentArea$MainContentArea$editControl$printHistory$HistoryRepeater$ctl40$linkEditSearch',''))S41 |
| S42 | TI visual N2 feedback OR AB visual N2 feedback | Search modes - Boolean/Phrase | 3,238 | [Edit](javascript:__doPostBack('ctl00$ctl00$MainContentArea$MainContentArea$editControl$printHistory$HistoryRepeater$ctl41$linkEditSearch',''))S42 |
| S43 | TI digital N2 feedback OR AB digital N2 feedback | Search modes - Boolean/Phrase | 97 | [Edit](javascript:__doPostBack('ctl00$ctl00$MainContentArea$MainContentArea$editControl$printHistory$HistoryRepeater$ctl42$linkEditSearch',''))S43 |
| S44 | TI feedback N2 strateg* OR AB feedback N2 strateg* | Search modes - Boolean/Phrase | 693 | [Edit](javascript:__doPostBack('ctl00$ctl00$MainContentArea$MainContentArea$editControl$printHistory$HistoryRepeater$ctl43$linkEditSearch',''))S44 |
| S45 | TI graph* N2 feedback OR AB graph* N2 feedback | Search modes - Boolean/Phrase | 221 | [Edit](javascript:__doPostBack('ctl00$ctl00$MainContentArea$MainContentArea$editControl$printHistory$HistoryRepeater$ctl44$linkEditSearch',''))S45 |
| S46 | TI adherence N2 feedback OR AB adherence N2 feedback | Search modes - Boolean/Phrase | 74 | [Edit](javascript:__doPostBack('ctl00$ctl00$MainContentArea$MainContentArea$editControl$printHistory$HistoryRepeater$ctl45$linkEditSearch',''))S46 |
| S47 | TI compliance N2 feedback OR AB compliance N2 feedback | Search modes - Boolean/Phrase | 42 | [Edit](javascript:__doPostBack('ctl00$ctl00$MainContentArea$MainContentArea$editControl$printHistory$HistoryRepeater$ctl46$linkEditSearch',''))S47 |
| S48 | S40 OR S41 OR S42 OR S43 OR S44 OR S45 OR S46 OR S47 | Search modes - Boolean/Phrase | 75,761 | [Edit](javascript:__doPostBack('ctl00$ctl00$MainContentArea$MainContentArea$editControl$printHistory$HistoryRepeater$ctl47$linkEditSearch',''))S48 |
| S49 | (MH "Medication Adherence") | Search modes - Boolean/Phrase | 0 | [Edit](javascript:__doPostBack('ctl00$ctl00$MainContentArea$MainContentArea$editControl$printHistory$HistoryRepeater$ctl48$linkEditSearch',''))S49 |
| S50 | TI medic* N2 compliance OR AB medic* N2 compliance | Search modes - Boolean/Phrase | 1,878 | [Edit](javascript:__doPostBack('ctl00$ctl00$MainContentArea$MainContentArea$editControl$printHistory$HistoryRepeater$ctl49$linkEditSearch',''))S50 |
| S51 | TI medic* N2 concordance OR AB medic* N2 concordance | Search modes - Boolean/Phrase | 72 | [Edit](javascript:__doPostBack('ctl00$ctl00$MainContentArea$MainContentArea$editControl$printHistory$HistoryRepeater$ctl50$linkEditSearch',''))S51 |
| S52 | TI medic* N2 adherence OR AB medic* N2 adherence | Search modes - Boolean/Phrase | 6,934 | [Edit](javascript:__doPostBack('ctl00$ctl00$MainContentArea$MainContentArea$editControl$printHistory$HistoryRepeater$ctl51$linkEditSearch',''))S52 |
| S53 | TI medic* N2 non-adherence OR AB medic* N2 non-adherence | Search modes - Boolean/Phrase | 601 | [Edit](javascript:__doPostBack('ctl00$ctl00$MainContentArea$MainContentArea$editControl$printHistory$HistoryRepeater$ctl52$linkEditSearch',''))S53 |
| S54 | TI medic* N2 taking OR AB medic* N2 taking | Search modes - Boolean/Phrase | 4,290 | [Edit](javascript:__doPostBack('ctl00$ctl00$MainContentArea$MainContentArea$editControl$printHistory$HistoryRepeater$ctl53$linkEditSearch',''))S54 |
| S55 | S49 OR S50 OR S51 OR S52 OR S53 OR S54 | Search modes - Boolean/Phrase | 12,371 | [Edit](javascript:__doPostBack('ctl00$ctl00$MainContentArea$MainContentArea$editControl$printHistory$HistoryRepeater$ctl54$linkEditSearch',''))S55 |
| S56 | TI ( perspectives or views or perceptions or attitudes or opinions or understanding or experience ) OR AB ( perspectives or views or perceptions or attitude or opinions or understanding or experience ) | Search modes - Boolean/Phrase | 1,694,580 | [Edit](javascript:__doPostBack('ctl00$ctl00$MainContentArea$MainContentArea$editControl$printHistory$HistoryRepeater$ctl55$linkEditSearch',''))S56 |
| S57 | S39 OR S48 | Search modes - Boolean/Phrase | 92,838 | [Edit](javascript:__doPostBack('ctl00$ctl00$MainContentArea$MainContentArea$editControl$printHistory$HistoryRepeater$ctl56$linkEditSearch',''))S57 |
| S58 | S55 AND S56 AND S57 | Search modes - Boolean/Phrase | 61 | [Edit](javascript:__doPostBack('ctl00$ctl00$MainContentArea$MainContentArea$editControl$printHistory$HistoryRepeater$ctl57$linkEditSearch',''))S58 |

**PubMed database search (Date: 14/02/2023)**

**
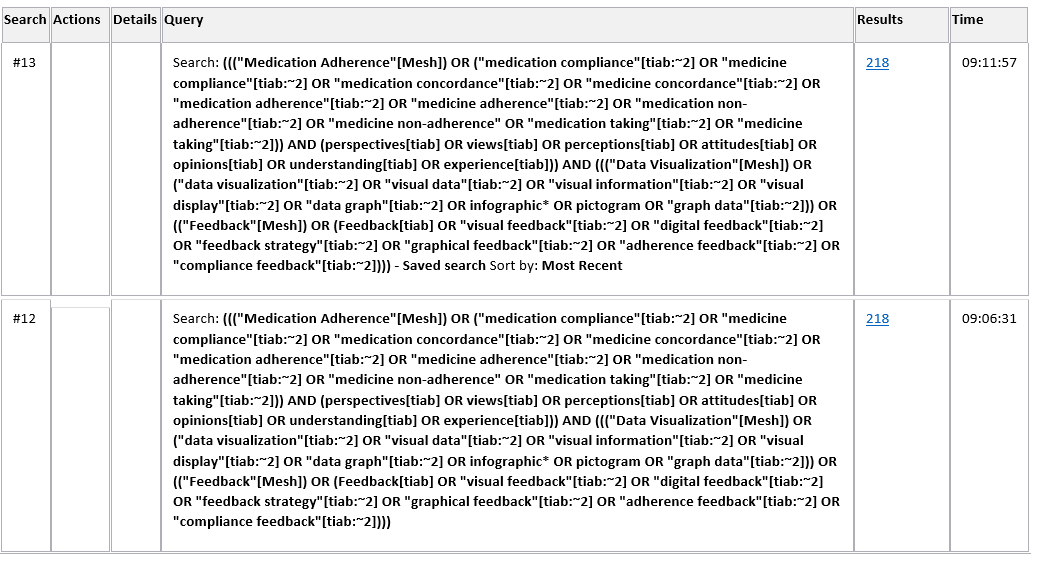
**


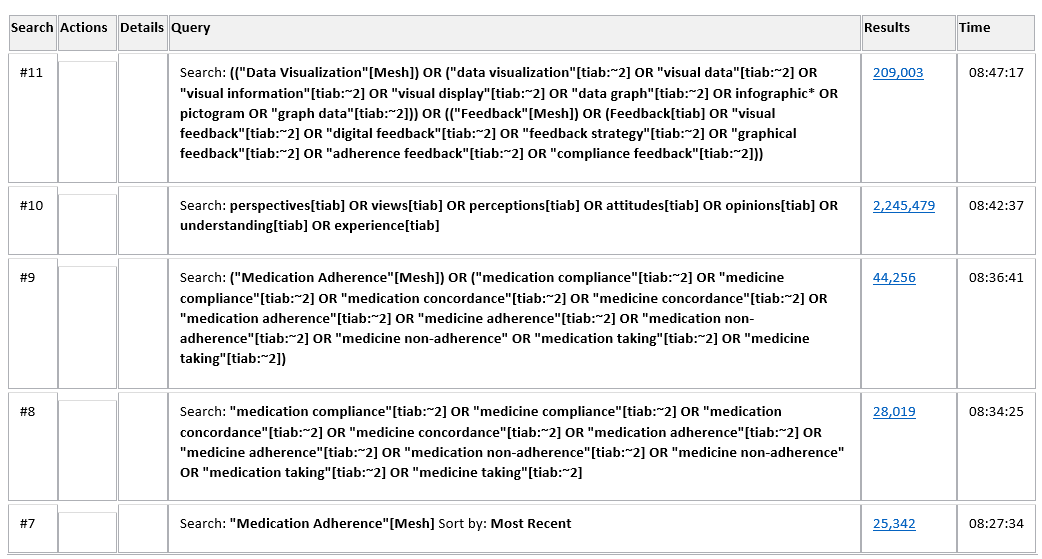


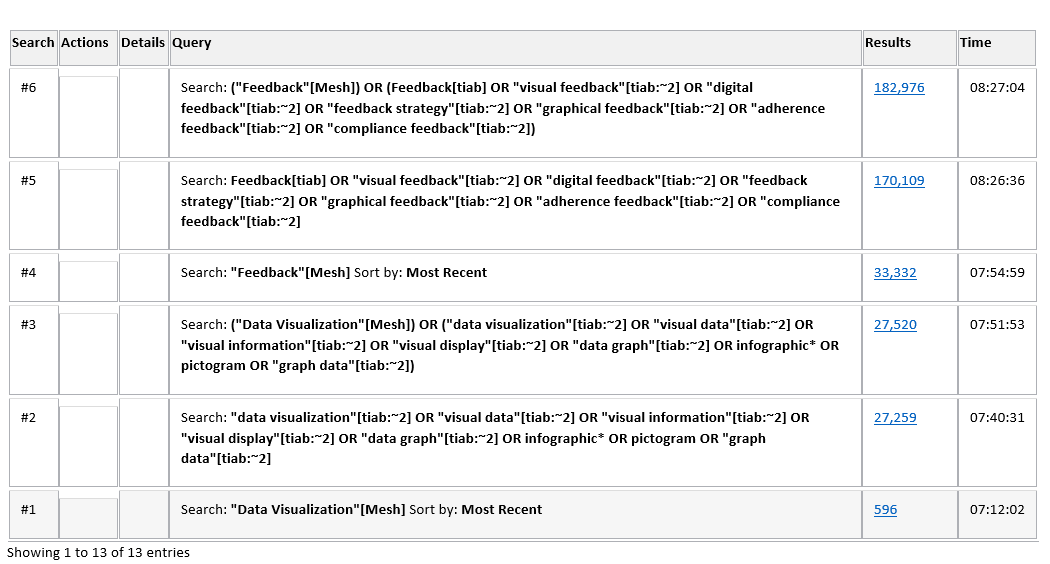


UPDATED DATABASE SEARCH

**Updated CINAHL database Search (Date:02/02/2025)**


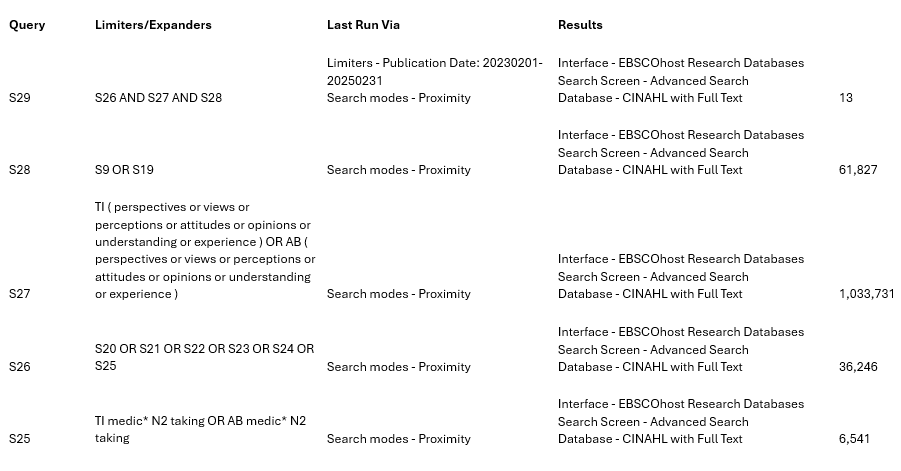


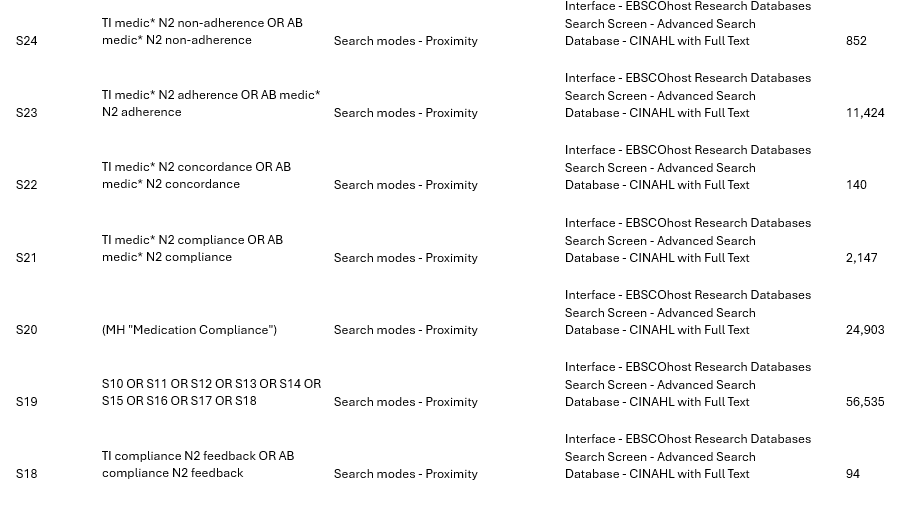


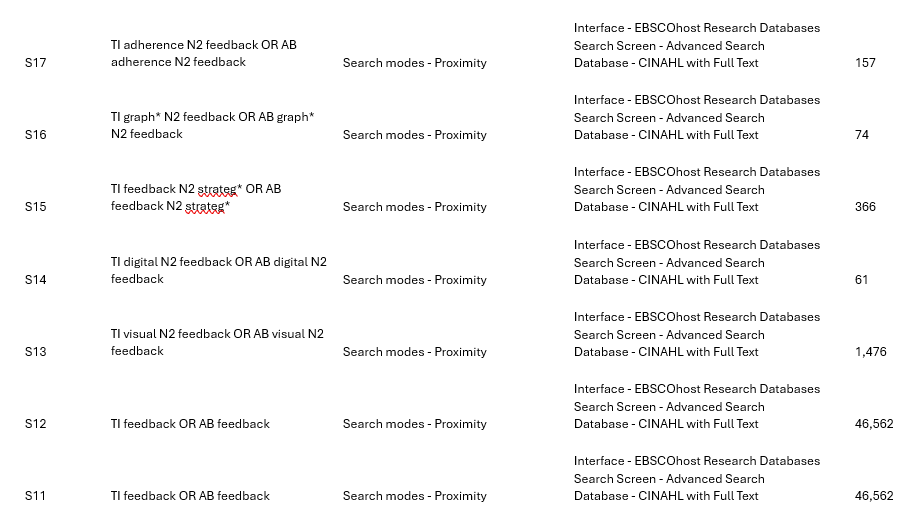


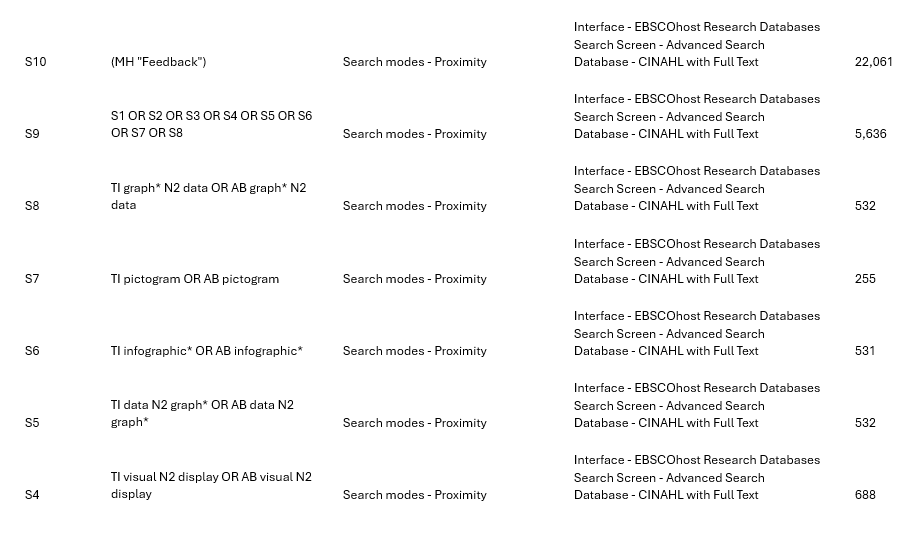

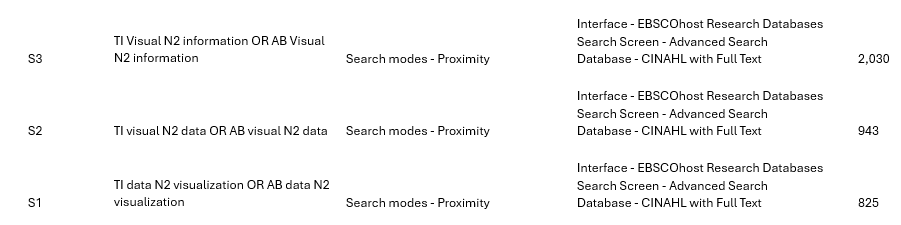


**Updated Embase database search (Date:05/02/2025)**

1 exp data visualization/ 4748

2 data visualization.tw. 3290

3 visual data.tw. 1113

4 visual information.tw. 12610

5 visual display.tw. 2197

6 data graph*.tw. 375

7 infographic*.tw. 1767

8 pictogram.tw. 536

9 graph data.tw. 392

10 1 or 2 or 3 or 4 or 5 or 6 or 7 or 8 or 9 25470

11 feedback.tw. 249372

12 visual feedback.tw. 6505

13 digital feedback.tw. 102

14 feedback strateg*.tw. 424

15 graph* feedback.tw. 95

16 adherence feedback.tw. 76

17 compliance feedback.tw. 26

18 11 or 12 or 13 or 14 or 15 or 16 or 17 249372

19 exp medication compliance/ 52683

20 medic* compliance.tw. 4188

21 medic* concordance.tw. 63

22 medic* adherence.tw. 26633

23 medic* non-adherence.tw. 2306

24 medic* taking.tw. 2255

25 19 or 20 or 21 or 22 or 23 or 24 65983

26 (perspectives or views or perceptions or attitudes or opinions or understanding or experience).tw. 3299217

27 10 or 18 273396

28 25 and 26 and 27 377

29 limit 28 to yr="2023 - 2025" 67

**Updated MEDLINE database search (Date: 02/02/2025)**


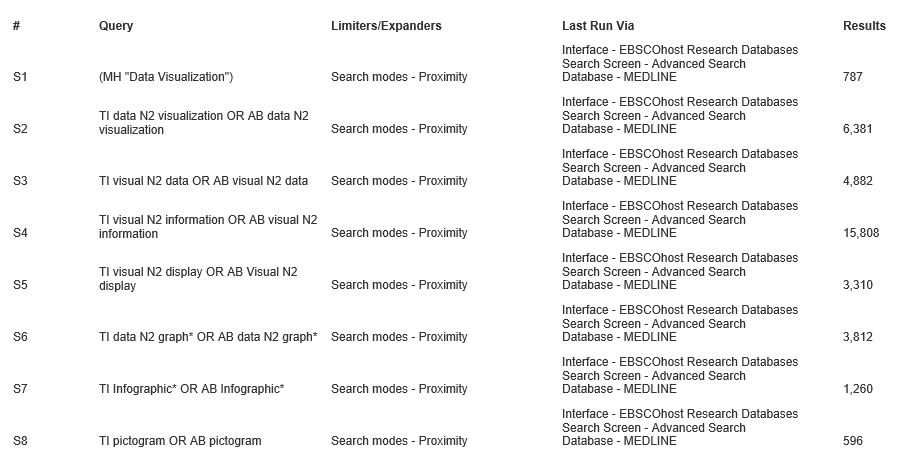

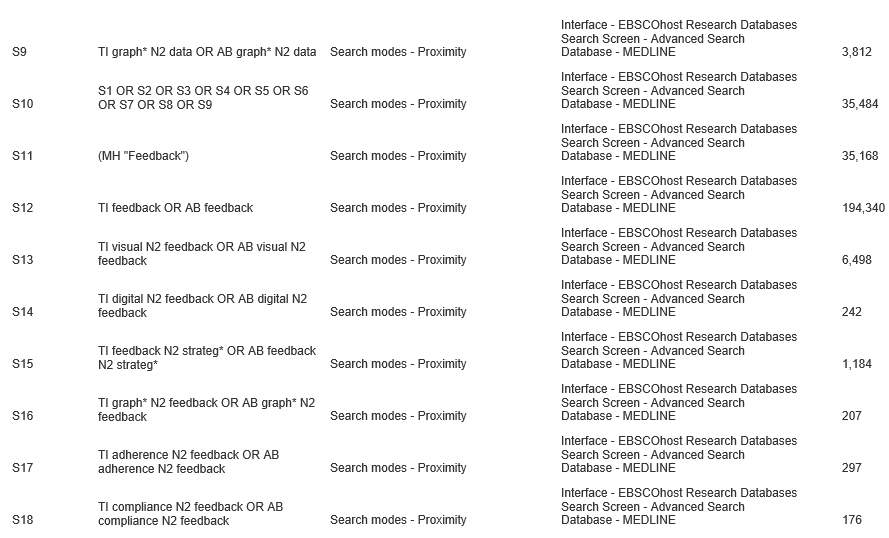


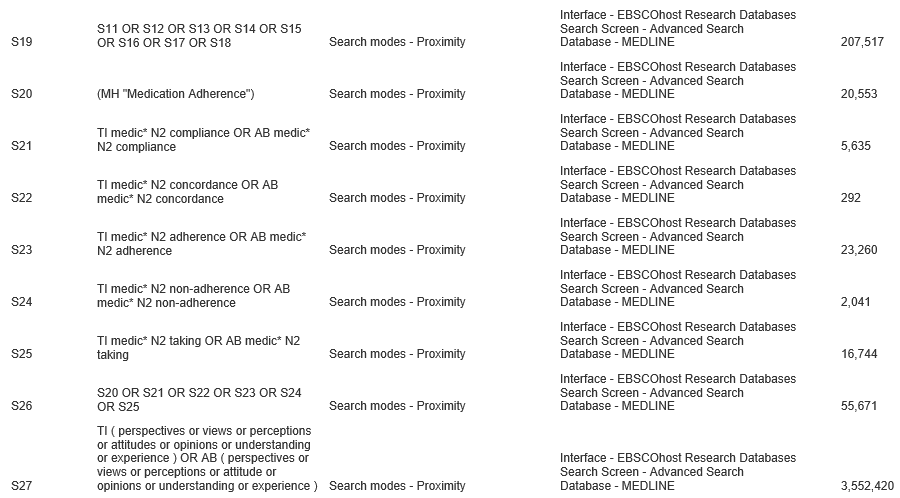


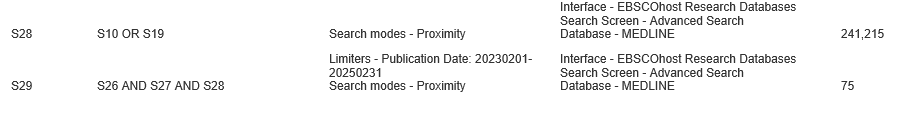


**Updated APA PsycInfo database search (Date: 03/02/2025)**


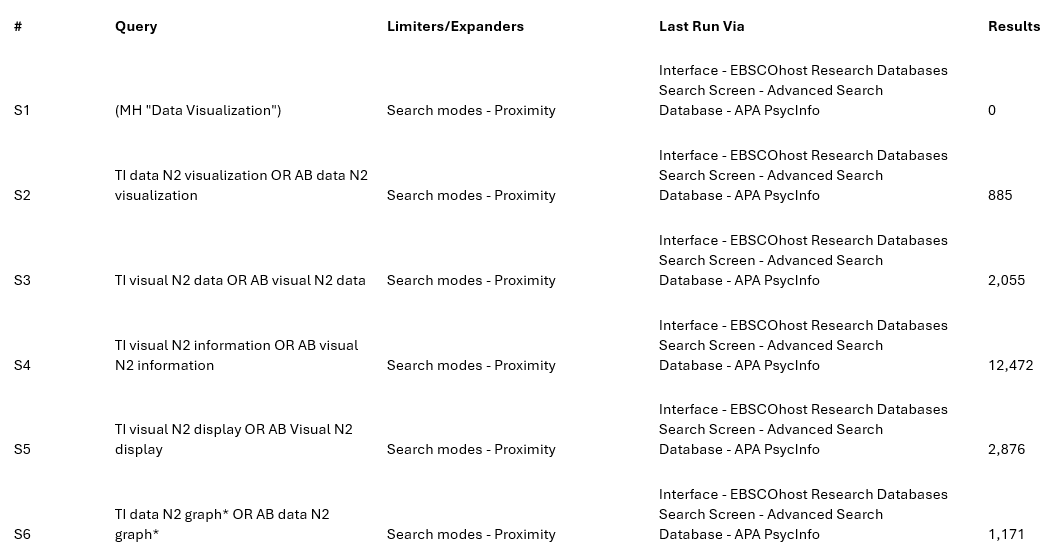

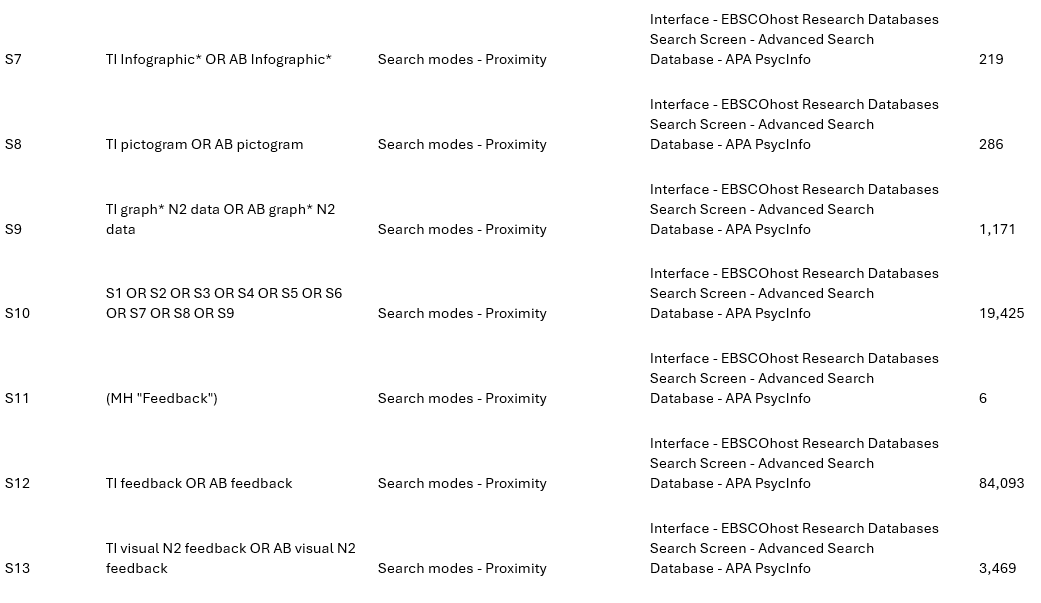


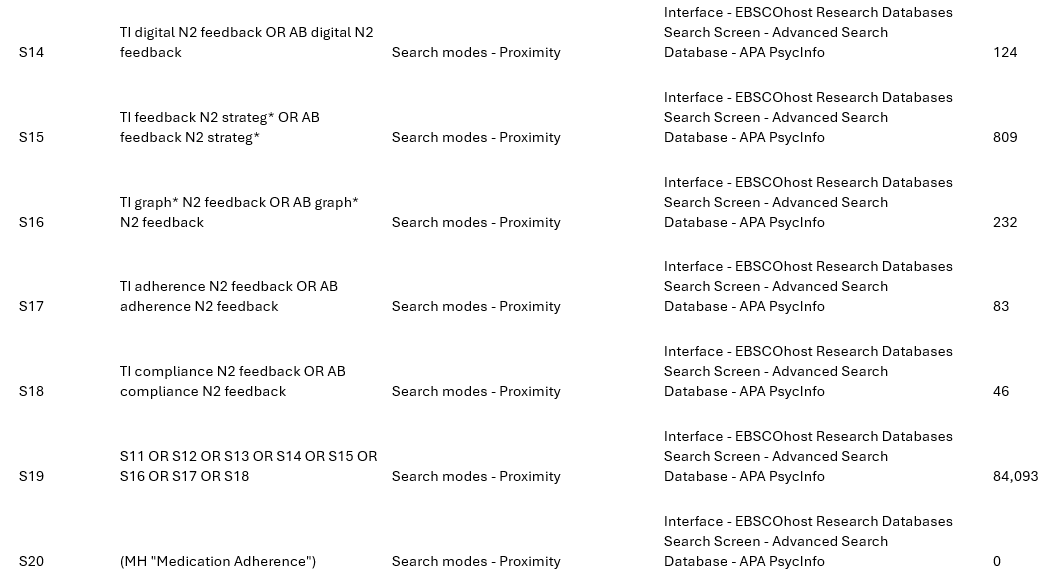


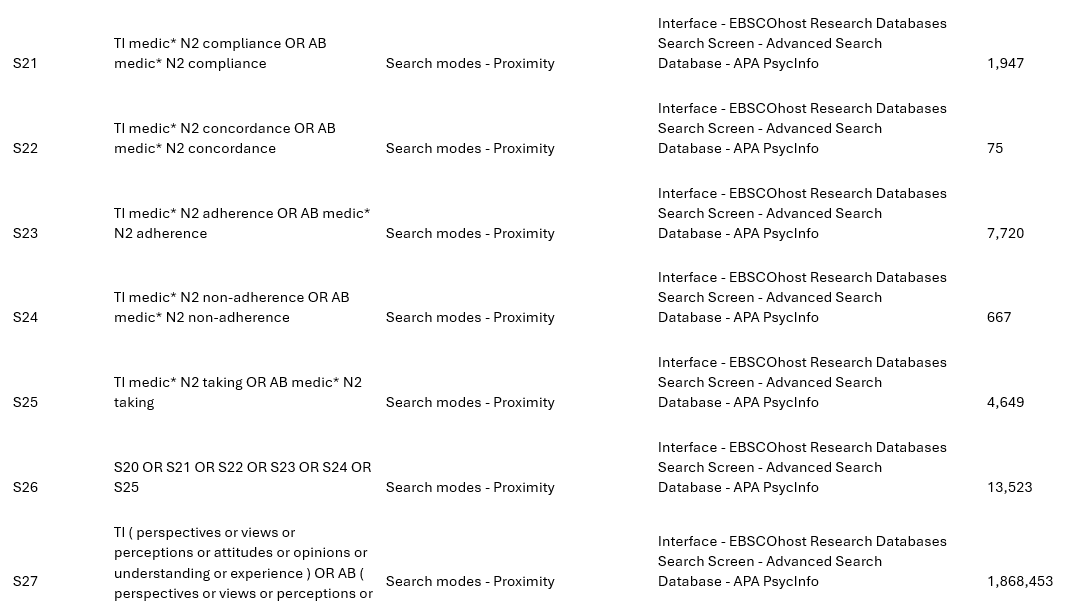

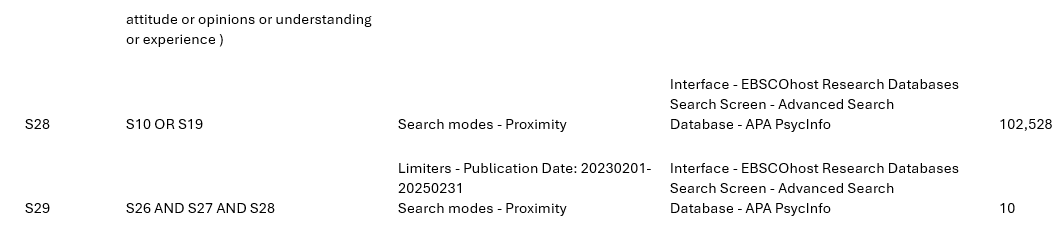


**Update PubMed Search (Date: 03/02/2025)**


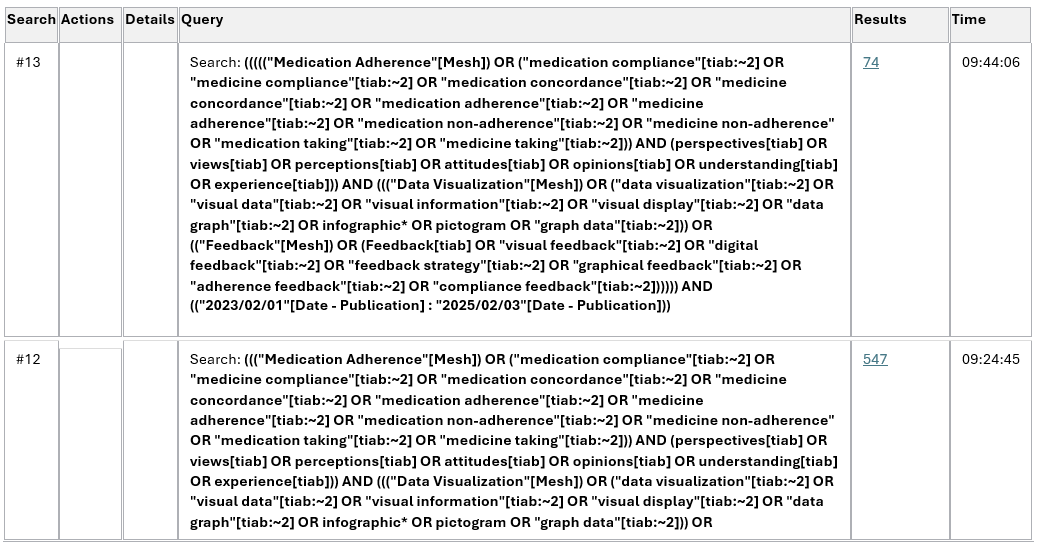


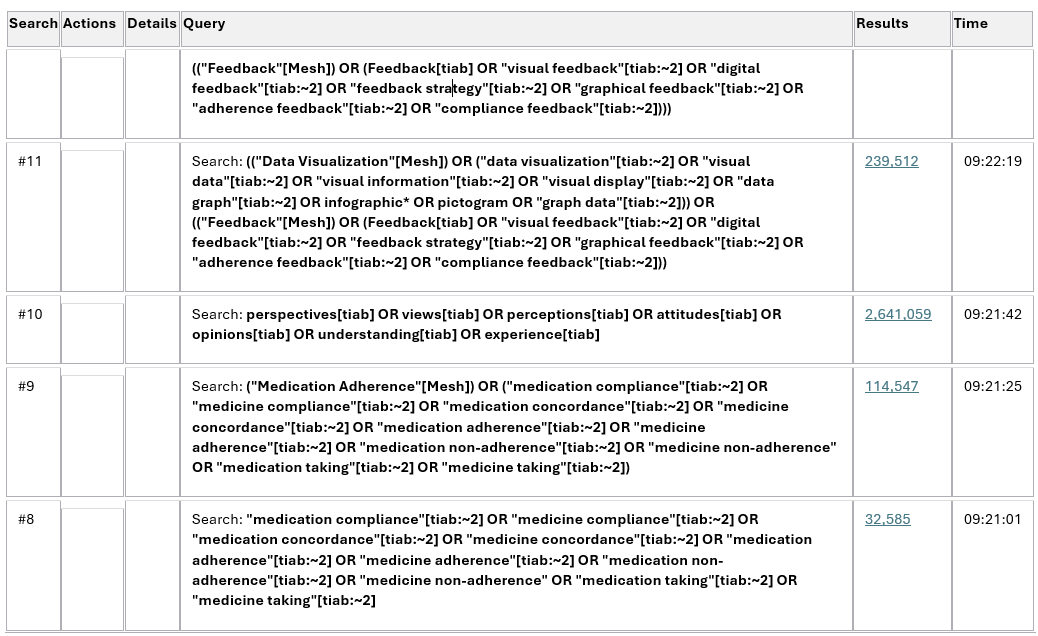


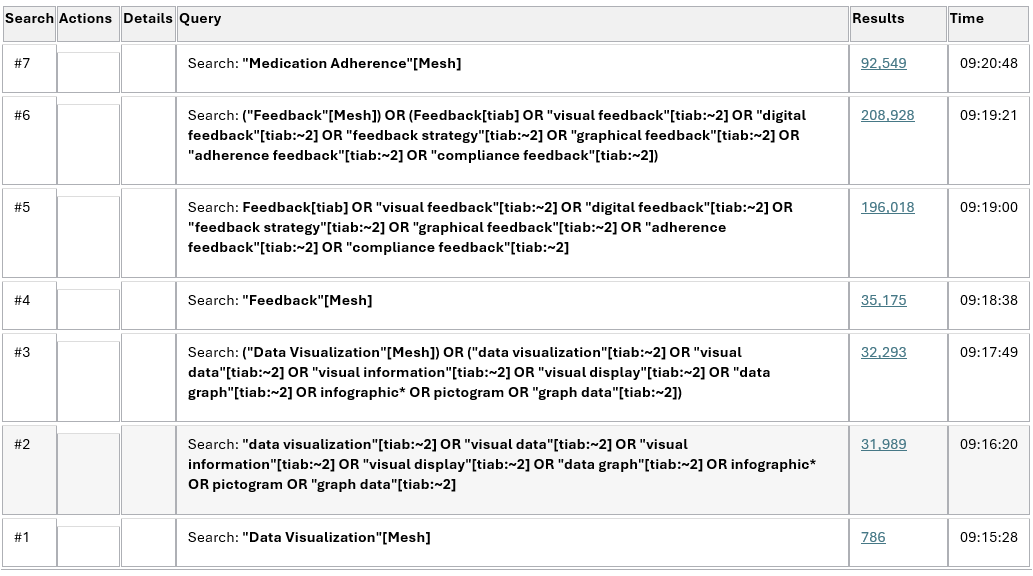

Supplement: Supplementary file 1 — Supplementary file1 (DOCX 2444 KB) [file 11096_2025_1958_MOESM1_ESM.docx]
